# Unveiling Distinct Proteomic Signatures in Complicated Crohn’s Disease That Could Predict the Disease Course

**DOI:** 10.3390/ijms242316966

**Published:** 2023-11-30

**Authors:** Laura A. Lucaciu, Radu Seicean, Alina Uifălean, Maria Iacobescu, Cristina A. Iuga, Andrada Seicean

**Affiliations:** 1 Department of Gastroenterology and Hepatology, “Iuliu Haţieganu” University of Medicine and Pharmacy, Croitorilor 19-21, 400162 Cluj-Napoca, Romania; lauraa.lucaciu@gmail.com (L.A.L.); andradaseicean@gmail.com (A.S.); 2 Department of General Surgery, First Surgical Clinic, “Iuliu Haţieganu” University of Medicine and Pharmacy, Clinicilor 3-5, 400006 Cluj-Napoca, Romania; rseicean@yahoo.com; 3 Department of Pharmaceutical Analysis, Faculty of Pharmacy, “Iuliu Haţieganu” University of Medicine and Pharmacy, Louis Pasteur 6, 400349 Cluj-Napoca, Romania; alina.uifalean@umfcluj.ro (A.U.); iugac@umfcluj.ro (C.A.I.); 4 Department of Proteomics and Metabolomics, MEDFUTURE-Research Centre for Advanced Medicine, “Iuliu Haţieganu” University of Medicine and Pharmacy, Louis Pasteur 4, 400349 Cluj-Napoca, Romania; 5 “Prof. Dr. Octavian Fodor” Regional Institute of Gastroenterology and Hepatology, Croitorilor Street No. 19-21, 400162 Cluj-Napoca, Romania

**Keywords:** Crohn’s disease, proteomics, disease progression, biomarkers, mass-spectrometry analysis, stricturing behavior, inflammatory bowel diseases

## Abstract

Crohn’s disease (CD) is characterized by a chronic, progressive inflammation of the gastrointestinal tract often leading to complications, such as strictures and fistulae. Currently, there are no validated tools anticipating short- and long-term outcomes at an early stage. This investigation aims to elucidate variations in protein abundance across distinct CD phenotypes with the objective of uncovering potential biomarkers implicated in disease advancement. Serum samples collected from 30 CD patients and 15 healthy age-matched controls (HC) were subjected to depletion of highly abundant proteins and to a label-free mass spectrometry analysis. Twenty-four proteins were shown to be significantly different when comparing CD with HC. Of these, WD repeat-containing protein 31 (WDR31), and proteins involved in the acute inflammatory response, leucine-rich alpha-2-glycoprotein (LRG1) and serum amyloid A1 (SAA1), were more abundant in the aggressive subgroup. Against standard biomarkers, a positive correlation between SAA1 and WDR31 and C-reactive protein (CRP) was found. In this study, a unique serum biomarker panel for aggressive CD was identified, which could aid in predicting the disease course.

## 1. Introduction

Crohn’s disease (CD) is a chronic, transmural inflammation of the gastrointestinal tract with variable phenotypes, affecting treatment choice and patient outcomes. Currently, treatment choice is based on the clinical presentation of the disease, which is highly heterogenous and can be confounded by delays in the diagnosis. Up to 30% of patients with CD have evidence of bowel damage at diagnosis, and half may undergo surgery within 20 years after their diagnosis [1]. Biomarkers indicative of the molecular progression of the disease that would anticipate the high variability in the prognosis that occurs between patients are therefore required to deliver optimal therapy.

Previous attempts to predict the disease course have focused on clinical parameters, but these criteria (including younger age at diagnosis, early steroid requirement, perianal involvement, and deep ulcers at endoscopy) lack specificity and are not useful for patient stratification [2]. Genetic association studies have led to subphenotypic stratification in IBD as well as to the discovery of multiple pathways driving the inflammatory response [2].

A serum proteomic analysis has yielded significant insight into aspects of IBD so far, from differentiating subtypes and stages of the disease [3,4] to predicting the disease course [5,6] and response to therapy [7]. The most used techniques for candidate biomarker discovery are mass spectrometry and bioinformatic techniques, which allow for the rapid screening and analysis of large numbers of proteins from a target sample [6]. However, none of the identified proteomic biomarkers have been implemented in daily clinical use, and early accurate prognostication is yet to be achieved.

In this study, we aimed to characterize the serum proteome of CD patients with different phenotypes (penetrating, stricturing, or inflammatory) in order to identify new biomarker candidates along with possible pathways involved in the development of these complications. We also investigated the association of protein abundance at baseline with conventional markers used in the clinic, such as C-reactive protein (CRP), fecal calprotectin (Fcal), and albumin (ALB).

## 2. Results

### 2.1. Baseline Characteristics of the Study Participants

Thirty patients with CD were included, fifteen with the inflammatory phenotype (B1) and fifteen with the complicated phenotype (B2B3), based on the criteria described in the Materials and Methods section. Patients in the B1 subgroup were older than patients in the B2B3 group. Patients with a stricturing or penetrating phenotype (B2B3, n = 15) had significantly higher HBI and SES-CD scores and received more frequent anti-TNFα medication; in total, 22/30 patients had anti-TNF treatment (Table 1). FCal (µg/g) showed higher values in patients with a stricturing or penetrating phenotype and they reported more frequently an IBD-related surgery than those with an inflammatory phenotype, but these findings were not statistically significant (Table 1).

At a 1-year follow up, no significant differences were observed between patients with different CD phenotypes with regards to the disease activity score (HBI), laboratory tests indicative of inflammation (C-reactive protein (CRP), albumin (ALB), fecal calprotectin (FCal)), and outcome (surgery, treatment escalation/change) (Table 2).

### 2.2. Serum Proteome Characterization

Over 6300 unique peptides corresponding to 781 proteins and protein families with at least one unique peptide were identified. After applying the criterion for inclusion in the analysis (please see Section 4, Materials and Methods; Section 4.4, Statistical Analysis), a total number of 770 proteins were further subjected to a biomarker analysis. The complete list of detected proteins considered for the analysis is shown in Supplementary Table S1.

#### 2.2.1. Deciphering Serum Proteome Group Patterns

**Figure 1:**
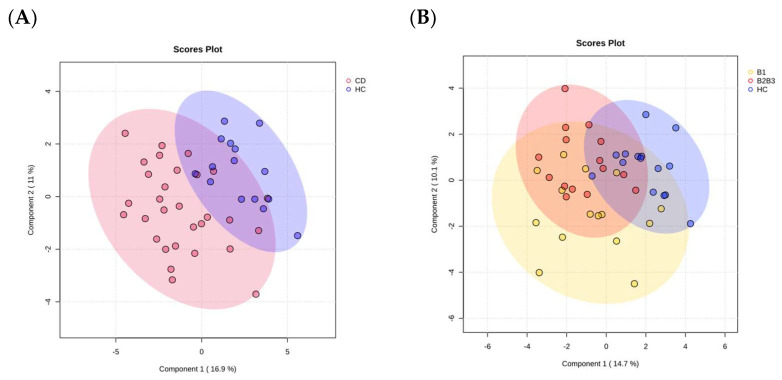
Sampling group clustering by the serum proteome of the CD and HC (**A**) and the B1, B2B3, and HC group (**B**).

As a proof of concept for our study, we performed a group clustering analysis. The identified proteins showed a favorable clustering of the analyzed groups. This was evidenced by applying a partial least squares discriminant analysis (PLS-DA), which showed separation based on the first three components: the CD and HC groups (Figure 1A), and the B1, B2B3, and HC groups (Figure 1B).

#### 2.2.2. Serum Proteome Alterations in Crohn’s Disease

**Figure 2:**
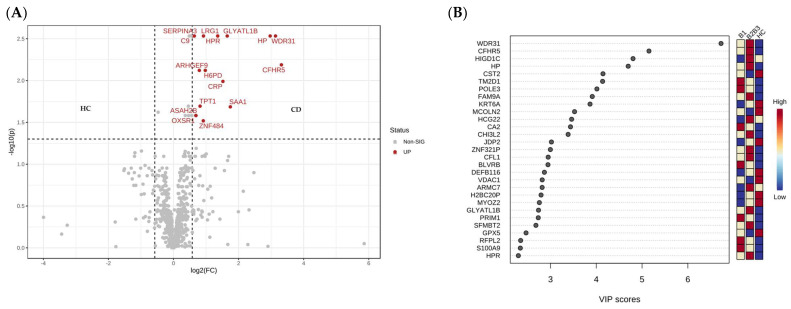
Volcano plot showing the significantly different serum proteins among the CD and HC groups (**A**). The VIP score plot corresponding to the PLS-DA analysis interrogating the serum proteome alterations in the stricturing and penetrating Crohn’s disease phenotypes (**B**).

In total, 24 proteins were shown to be significantly different when comparing CD (B1 and B2B3 taken together as a group) with HC. Among them, 16 had a fold change greater than 1.5 and all were higher in the CD group (Figure 2A); among them, Complement Factor H-related protein (CFHR), WD repeat-containing protein 31 (WDR31), Haptoglobin (HP), and serum amyloid A1 (SAA1) were evidenced. The PLS-DA analysis was also carried out and the CD vs. HC discriminating proteins were evidenced (Supplementary Table S2, Supplementary Figure S1).

#### 2.2.3. Serum Proteome Alterations in the Aggressive Crohn’s Disease Phenotype

By applying a one-way analysis of variance (parametric ANOVA), 29 proteins were shown to be significantly different between the three groups, and Figure 2B shows the corresponding PLS-DA variable importance projection (VIP) scores plot. Table 3 shows the proteins and the fold changes between each group comparison. The 29 proteins are listed in Supplementary Table S3 together with the fold changes and the VIP scores for the three components of the PLS-DA analysis.

### 2.3. Biomarker Signature Scouting toward Disease Course Prediction

In pursuit of biomarker exploration for the prediction of Crohn’s disease progression, we conducted an analysis utilizing the Area Under the Curve (AUC) metric. Reflecting the extent of differentiation between the two Crohn’s disease phenotypes based on proteome biomarkers, the AUC was computed for proteins displaying statistically significant variations and a fold change exceeding 1.5.

To distinguish between the B2B3 and B1 groups, we examined three proteins: leucine-rich alpha-2-glycoprotein (LRG1), WD repeat-containing protein 31 (WDR31), and serum amyloid A-1 protein (SAA1). All three proteins individually exhibited AUC values exceeding 0.7. When considered together as a three-biomarker panel, these proteins yielded an AUC greater than 0.7, as determined through the application of the PLS-DA algorithm.

**Figure 3:**
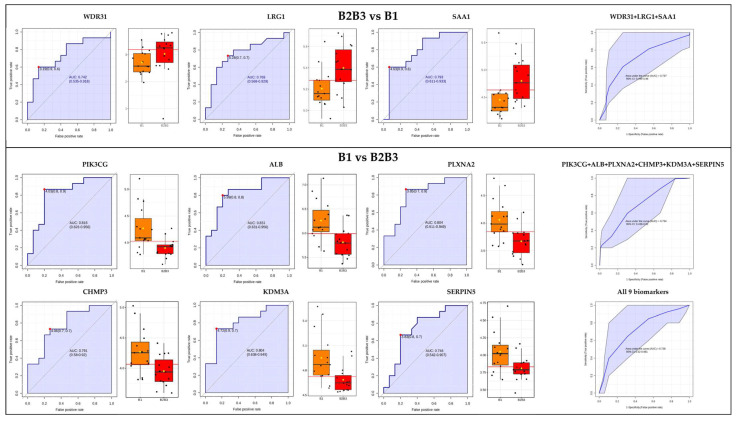
Proteins showing discriminatory potential between the Crohn’s disease phenotypes based on AUC analysis. Red dot value = cut-off value (log10-normalized) with highest specificity, sensibility in brackets, AUC = Area Under the Curve, 95% confidence band, CI = confidence interval.

When comparing the B1 group with the B2B3 group, we identified a set of six proteins: phosphatidylinositol 4,5-bisphosphate 3-kinase catalytic subunit gamma isoform (PIK3CG), albumin (ALB), Plexin-A2 (PLXNA2), charged multivesicular body protein 3 (CHMP3), lysine-specific demethylase 3A (KDM3A), and plasma serine protease inhibitor (SERPINA5). These proteins demonstrated substantial AUC values both individually and as potential components of a multi-marker panel. Values and figures are presented in the figure below (Figure 3).

### 2.4. Proteome Correlation with Clinical Biomarkers

In the quest for correlating our proteome findings with the clinical biomarkers, Spearman rank correlation was used. We explored the relationship between the nine highlighted serum proteins from the previous analysis and disease phenotype, as well as the clinical biomarkers HBI, CRP, ALB, and FCal (Table 4).

## 3. Discussion

As treatment goals in Crohn’s disease shifted from clinical remission to mucosal and transmural healing, the need for novel biomarkers to assess fibro-inflammatory processes that lead to a poor prognosis is increasing. Deep molecular profiling of tissue and blood with multi-modal omics technologies (transcriptomics, metagenomics, metabolomics, and proteomics) is bringing new insight in patient stratification. We have therefore compared serum protein profiles between different CD phenotypes through a hypothesis-free approach, with the aim of identifying specific compounds that might aid in predicting the disease course.

This study reveals for the first time the different proteomic signatures between the non-stricturing, non-penetrating B1 group, and the stricturing and penetrating Crohn phenotypes, the B2B3 group, based on a three-protein panel. This serum three-protein panel is characterized by higher levels of LRG1, WDR31, and SAA1 in the stricturing and penetrating group. The three proteins are known to be associated with the acute inflammatory response, signal transduction, autophagy, and apoptosis.

Furthermore, by investigating the relationship between the serum proteins that showed potential biomarker value and the diagnosis and the clinical biomarkers HBI, CRP, ALB, and FCal, a good correlation was found between two proteins, namely SAA1 and WDR31, and CRP, but not with FCal.

CRP is a well-established marker for estimating inflammation and disease activity in CD [8]. A Norwegian population-based study from the IBSEN cohort showed that persistently elevated CRP concentrations 1 year after the diagnosis could predict a need for abdominal surgery in CD patients [9]. Also, persistently elevated CRP levels, even in patients in remission, may predict poor outcomes such as hospitalization and intestinal resection during follow up [10]. This association supports the role of SAA1 as a potential biomarker for an aggressive disease course.

Our findings are consistent with previous studies [5,6]; a pilot study by Townsend et al. showed that the stricturing CD phenotype is distinguishable from non-stricturing CD and ulcerative colitis via a proteomic analysis with up to 80% accuracy [6]. Furthermore, aiming to predict CD behavior, Piras et al. reported a differential display of several serum proteins in early-stage CD, such as overexpression of inflammatory proteins and complement 3 chain C [5]. A study analyzing a multi-protein panel in newly diagnosed IBD showed that a pre-selected panel of proteins can not only differentiate IBD from controls but also predict the disease course and need for treatment intensification [11]. More recently, a study of 201 CD patients using pre-diagnosis samples collected at multiple timepoints highlighted a set of 22 protein biomarkers associated with complicated CD [12], of which included SAA1, which is in keeping with our study findings.

LRG, a 50 kDa glycoprotein including eight leucine-rich repeat domains has been reported to be a novel surrogate biomarker for inflammation in inflammatory bowel disease [13]. In Japan, LRG was approved as a novel biomarker to assess disease activity in patients with ulcerative colitis in 2020. A prospective study of 267 IBD patients (203 ulcerative colitis cases and 64 CD cases) comparing the levels of LRG with CRP and FCal against clinical and endoscopic disease activity has shown similar detectability of endoscopic inflammation between FCal and LRG in CD patients [14]. Although the precise function of LRG remains unclear, LRG is secreted during the acute phase of inflammation in response to inflammatory cytokines such as IL-6, TNF-α, and IL-22, which play a crucial role in IBD pathogenesis and are elevated in patients with active disease [15,16,17]. As such, in active IBD, an excess of inflammatory cytokines (IL-1β, Il-6, TNF-α) could induce LRG expression and extracellular secretion in the peripheral blood, with the levels being proportionate with disease activity. Furthermore, an elevation of LRG might promote inflammation in IBD by enhancing the differentiation of Th-17 cells and through its effect in increasing angiogenesis, as previously reported [18]. In this context, our study is the first to show a possible role for LRG in discriminating a more severe outcome as well, as persistent inflammation or failure to control active inflammation in IBD may lead to an increasing risk of complications such as strictures and fistulae [1].

WDR31 is a member of the WD40 repeat proteins family, involved in signal transduction, regulation of transcription, regulation of the cell cycle, autophagy, and apoptosis [19]. Mutations of this family of proteins have been involved in various disorders (neurological, cancers, endocrine, and ciliopathies) [20]. Like WDR30 (ATG16L1), a known CD susceptibility gene in Western CD patients and also involved in autophagy, WDR31 was recently described as a candidate gene for CD susceptibility in Japanese patients [19,21]. However, the function of WDR-31 is currently unknown and no correlation with CD phenotype has been reported so far. Its involvement in IBD pathogenesis could be explained by a dysregulated function of autophagy-related genes leading to inflammatory, immune, and metabolic disorders [22]. Autophagy has a crucial role in (1) regulating intestinal barrier function via inducing lysosomal degradation of the tight junction protein claudin 2 (CLDN2), thus decreasing intestinal permeability [23]; (2) modulating cytokine-induced programmed cell death in the intestinal epithelium, thus limiting intestinal inflammation [24]; (3) maintaining gut microbiota composition [25]; and (4) inhibition of inflammasome activation and subsequently controlling intestinal inflammation as shown by the protective effect on DSS-induced colitis in mice by inducing autophagy [26]. According to our findings, WDR31 could also be associated with a more complicated clinical disease phenotype, and this study is the first to report this association.

The SAAs are a family of acute phase response proteins that have been associated with gut microbial ecology and inflammation [27,28]. In healthy individuals, the plasma level of SAA is very low; however, it promptly increases up to 1000-fold in response to inflammation, trauma, or viral infections [29]. Recently, SAA has been demonstrated to participate in immune regulation, especially T-cell immunity; SAA can regulate innate and adaptive immunity [28]. In IL2−/− and IL-10− mouse models of colitis, SAA levels correlated with disease severity, as well as tumorigenesis in a mouse model of colon-associated cancer [14]. In IBD, SAA can exert an important influence on the intestinal mechanical barrier, immune barrier, and microbiota by inducing cell differentiation and enhancing intestinal antibacterial effects; SAA1/2 has a proinflammatory effect, whereas SAA3 is more protective of the gut epithelium [30]. A high SAA1 in IBD patients is indicative of active endoscopic and histologic inflammation and could serve as a surrogate marker of disease activity in those patients where CRP is not upregulated, as also shown by our analysis [13]. In our study, SAA1 proved to discriminate between complicated and inflammatory CD. This is the first time to our knowledge that a possible link between SAA1 levels and CD behaviors has been reported.

Proteins that were more abundant in the B1 group as compared to B2B3 have either shown involvement in IBD pathogenesis or have raised interest as potential biomarkers. Protein kinases play a crucial role in pathogenesis of IBD, by regulating chemokine-mediated recruitment and activation of immune cells [27]. Plexins are one of the most representative semaphorine receptors, with involvement in various immune disorders, such as rheumatoid arthritis, multiple sclerosis, and allergy; their specific role in IBD is currently unknown [28]. Also, not surprisingly, albumin, a biomarker that, when downregulated, correlates with malnutrition, high inflammatory burden, and poor prognosis, was higher in the inflammatory group of our study [31]. Serine protease inhibitors (serpins) are involved in the host–gut-microbiota interaction and may have a role in modulating the inflammatory response and underlying proteolytic pathways [32,33], therefore exhibiting a protective effect. Although previous studies have shown a decrease in serpines in active-IBD patients, in our study, the levels of SERPINA 5 were upregulated only in the inflammatory phenotype group. Furthermore, the levels of non-abundant proteins were not investigated in this study.

Our serum global proteome profiling study was based on a powerful data independent acquisition tool, namely high-definition mass spectrometry. This technique is widely used in many other biomarker discovery studies with well-acknowledged results and was also previously applied with success by our research group [34,35].

So far, our results are based only on a small number of patient samples and a single time-point analysis. Furthermore, we focused our analysis on a non-invasive biospecimen, serum, and proteins exhibiting substantial changes in mass abundance, namely a fold change greater than 1.5, to enhance the likelihood of successful detection and validation through an enzyme-linked immunosorbent assay, ELISA, which could be the next approach towards validation and clinical implementation. However, this approach may have overlooked low-abundance proteins indicative of a fibro-penetrating phenotype.

In summary, the stricturing and penetrating Crohn’s disease phenotype is characterized by distinct proteomic signatures, evidenced by elevated serum levels of WDR31, LRG1, and SAA1. To further establish the utility of these proteins as potential biomarkers and refine their clinical applicability, it is imperative to conduct larger-scale studies. Ongoing investigations by our group and collaborative partners with a more extensive participant cohort will be crucial for validating and solidifying the reliability of these protein markers. These endeavors are pivotal steps toward enhancing the clinician’s ability to identify individuals at risk of developing complications associated with Crohn’s disease.

## 4. Materials and Methods

### 4.1. Study Participants and Sampling

This was a cross-sectional, observational, analytical case–control study. Adult subjects with an established diagnosis of CD (n = 30), undergoing regular clinic follow up or hospitalization at a tertiary care center, namely the “Prof. Dr. Octavian Fodor” Regional Institute of Gastroenterology and Hepatology Cluj-Napoca, Romania, were prospectively recruited between 2016 and 2018, according to classical diagnosis criteria [36]. We selected patients in this cohort with CD who had either confirmed strictures or penetrating disease at the time of inclusion in this study, and an equal number of patients with persistent inflammation but no strictures or fistulae. Clinical management and decisions on diagnostic tests and medication were at the discretion of the treating physician. Blood samples for the proteomics analysis, inflammatory biomarkers (CRP, ESR, ALB), and fecal samples for FCal measurement were collected during admission as part of hospital protocol. Serum samples for the proteome analysis were aliquoted and stored at −80 °C.

Baseline demographic data, disease characteristics and phenotype, as well as the type and duration of IBD treatment (aminosalicylates, corticosteroids, immunosuppressive and biologic agents) were recorded. The Montreal classification system was used to assess the disease phenotype at baseline [5]. Disease location was described as follows: L1 (ileal), L2 (colonic), L3 (ileo-colonic), and L4 (upper gastrointestinal tract involvement proximal to the ligament of Treitz). Disease behavior was defined as B1 (inflammatory), B2 (stricturing), and B3 (penetrating), with a P modifier for concomitant perianal disease.

Classification was based on endoscopic and imaging data (computed tomography (CT) and/or magnetic resonance imaging (MRI) and intestinal ultrasound (IUS)) within 6 months of inclusion in this study, as follows: for B1 disease, evidence of mural/mucosal hyperenhancement only, persistent luminal narrowing with pre-stenotic dilatation in the case of B2 disease, or intra-abdominal fistulae leading to abscesses or fistulas to an adjacent organ, but excluding the vagina or perianal region. The endoscopic activity was assessed by calculating the Simplified Endoscopic Activity Score for Crohn’s Disease (SES-CD) [6].

CD subjects were followed up for 1 year to assess disease progression with clinical, standard biochemical tests and imaging techniques. Outcomes were defined as follows: clinical remission assessed by (1) HBI score, (2) need for surgery and (3) therapy modification (including cessation, escalation, or switch to another agent), and (4) a development of any B2 or B3 complication in the B1 group.

The control group consisted of 15 subjects referred to our center for outpatient colonoscopy. They were selected from outpatients who had a macroscopically normal colon and negative fecal and serum inflammatory biomarkers fecal calprotectin (FC), C-reactive protein (CRP), and eritrocite sedimentation rate (ESR), where available.

Patients that had a suspected or confirmed diagnosis of indeterminate colitis, infectious colitis, or malignancy, patients that were pregnant at admission, or those who expressed their refusal to participate were excluded from the study.

The study was conducted according to the guidelines of the WMA Declaration of Helsinki and approved by the Ethics Committee of the study center (decision number 16265/2016). Written informed consent was sought from all participants prior to inclusion and sample collection.

### 4.2. Sample Preparation for Proteomics Analysis

Serum was collected in tubes containing serum separator gel (BD Vacutainer, Franklin Lakes, NJ, USA) and was prepared according to the manufacturer’s instructions. Aliquots of serum were immediately stored at −80 °C until analysis.

#### 4.2.1. Depletion of Six Highly Abundant Serum Proteins

Serum albumin, immunoglobulin gamma, immunoglobulin alpha, serotransferrin, haptoglobin, and alpha-1-antitrypsin were depleted by using multi-affinity chromatography (MARS6-human) (Agilent Technologies, Waldbronn, Germany) following the manufacturer’s protocol. After depletion, samples were concentrated with trichloroacetic acid precipitation (final concentration: 15%) of the residual protein fraction, and the resulting pellet was resuspended in a urea/thiourea buffer (8/2 M) (VWR, Radnor, PA, USA) as previously carried out [35,37]. Subsequently, samples were stored at −80 °C until use.

#### 4.2.2. Proteolytic Digestion by Trypsin

Sample protein concentration was determined by using the microplate Bradford Assay (Invitrogen, Waltham, MA, USA) with bovine serum albumin as standard protein. In total, 4 μg of each protein sample was subjected to reduction with dithiothreitol (2.5 mM, 30 min at 37 °C), alkylation with iodoacetamide (10 mM, 15 min at 37 °C), and proteolytic digestion by trypsin (Merck KGaA, Darmstadt, Germany) at a 1:25 protease-to-protein ratio (overnight at 37 °C). In total, 1% acetic acid was used to stop digestion, and desalting of peptides was performed using an Oasis HLB 96-well μElution plate (Waters Corporation, Milford, MA, USA) following the manufacturer’s protocol. Lyophilized peptides were dissolved in 0.1% formic acid to a final concentration of 0.1 μg/μL prior to injection.

### 4.3. Proteome Profiling with Mass Spectrometry

#### 4.3.1. Protein Identification and Quantification with Nano-LC-HDMS^E^

Peptides (300 ng) were separated on an ACQUITY UPLC^®^ M-Class HSS T3 column (Waters Corporation, Milford, MA, USA) within 120 min with a non-linear gradient of 5% to 85% acetonitrile and 0.1% formic acid at a flow rate of 300 nL/min. An online coupled traveling wave ion-mobility-enabled hybrid quadrupole orthogonal acceleration time-of-flight mass spectrometer (SYNAPT G2-Si HDMS, Waters Corporation, Milford, MA, USA) was used to detect eluting peptides as previously performed. For data acquisition, the independent acquisition mode was employed (a programmed feature for parent and product ion measurement by switching between low energy (MS) and elevated energy (MS^E^)) and collision voltage ramping was set as a default. Samples were measured in two technical replicates and raw data were acquired using MassLynx™ Software Version 1.74.2662 (Waters Corporation, Milford, MA, USA). Detailed settings can be found in the Supplementary Methods.

#### 4.3.2. Database Search

LC–HDMS^E^ data were processed as previously reported [5]. In brief, Progenesis QI (v2.0, Waters Corporation, Milford, MA, USA) was used for automated peak picking and chromatogram alignment. The software built-in search engine was used for a spectra search using a Uniprot/Swissprot database (2022) limited to human entries (20,361) and the following parameters were set: enzyme specificity—trypsin (a maximum of 1 missed cleavage was allowed); carbamidomethylation of cysteine was set as fixed modification; and oxidation of methionine was set as a variable modification. Search tolerance parameters were as follows: false discovery rate < 4%, and proteins were considered for a further analysis only if the ion matching requirements were passed of fragments/peptide ≥ 2, fragments/protein ≥ 5, and peptides/protein ≥ 1. Peptide identifications were restricted to absolute mass error < 10 ppm, sequence length > 5, and score > 5 as previously carried out [35,37]. Protein relative quantification was performed on the summed peptide abundance by using only peptides, which have no conflicting protein identification.

### 4.4. Statistical Analysis

For the dataset capturing study participants’ characteristics at study entry and 1-year follow up (Table 1 and Table 2): The dataset’s normality was assessed using the Shapiro–Wilk Test, which confirmed non-normality (*p* > 0.05), leading to the use of non-parametric tests without normalization. Quantitative data were reported as the median [Q1 to Q3] and {minimum to maximum}, where Q1 is the 25th percentile and Q3 is the 75th percentile. The scores (HBI and SES-CD) were reported as the median [Q1 to Q3]. The differences between two groups (B1 vs. B2B3, CD vs. HC) were evaluated with the Mann–Whitney test while those between three groups (B1 vs. B2B3 vs. HC) were tested with the Kruskal–Wallis test. Qualitative data were summarized as reports (number of subjects with a specific characteristic/number of patients in the group) and differences were tested with Fisher’s exact test. In the evaluation of clinical and paraclinical outcomes, we applied two-tailed tests and a p-value less than 0.05 was considered statistically significant when two groups were compared, respectively, and at less than 0.017 when three groups were compared.

For the dataset capturing the global proteome profiling: Proteome data, inherently following a normal distribution, were exported from ProgenesisQI for proteomics after the software’s default normalization process at the protein level. Subsequently, a minimum of 70% valid values filter was applied to each patient group, and an abundance average was computed between the two technical replicates. The resulting matrix was imported into MetaboAnalyst 5.0 (https://www.metaboanalyst.ca). The identified missing values were imputed using estimated values determined through the k-nearest neighbors (KNN) algorithm on a feature-wise basis. Finally, a log10 transformation was applied before conducting the statistical analysis. Sampling group clustering was tested with a partial least squares discriminant analysis (PLS-DA) and discriminatory proteins were evidenced by PLS-DA variable importance projection (VIP) scores.

Since the proteomic data had a normal distribution, parametric tests were applied. Statistical significance was tested with the t-test and ANOVA with Tukey’s Honest Significant Difference (HSD) test, and the cut-off value for significance was set to *p* < 0.05. The fold change was calculated as the ratio of two group means. A significance cut-off level was set to fold change = 1.50. Biomarker performance was evaluated with the Area Under the Curve (AUC) and AUC PLS-DA algorithm, and the cut-off value was set to AUC = 0.7. Proteome correlation with clinical biomarkers was assessed with Spearman’s rank correlation and the significance level was set to *p* > 0.6. All statistical analyses were performed using default settings of the MetaboAnalyst 5.0 online omics data analysis platform.

## Figures and Tables

**Table 1:** Characteristics of the study participants at inclusion in the study.

Characteristic	Crohn’s Disease B1 (n = 15)	Crohn’s Disease B2B3 (n = 15)	*p*-Value *
Age, years	39 [31 to 45.5] {24 to 57}	31 [23.5 to 35] {18 to 44}	0.018
Male, no/n	7/15	8/15	0.715
Smoking			0.959
Current	3/15	3/15	
Former	4/15	5/15	
Never	5/15	5/15	
Unknown	3/15	2/15	
Baseline CRP (mg/dL)	0.4 [0.4 to 2.8] {0.31 to 4.48}	1 [0.4 to 5] {0.29 to 13.47}	0.237
Baseline ALB (g/dL)	4 [3.9 to 4.1] {0.85 to 5.1}	3.7 [3.2 to 4.1] {2.5 to 5.6}	0.351
Baseline FCal (µg/g)	200 [155 to 380] {20 to 860}	600 [300 to 910] {15 to 2300}	0.059
Montreal age (A)			0.636
A1 (<17 years)	1/15	3/15	
A2 (17–40 years)	12/15	10/15	
A3 (>40 years)	2/15	2/15	
Montreal location (L)			0.532
L1 (Terminal ileum)	6/15	3/15	
L2 (Colon)	4/15	4/15	
L3 (Ileo-colon)	5/15	7/15	
L4 (Upper GI)	0/15	1/15	
Montreal behavior (B)			not applicable
B1 (non-stricturing, non-penetrating)	15/15	0/15	
B2 (stricturing)	0/15	8/15	
B3 (penetrating)	0/15	3/15	
B2 + B3	0/15	4/15	
Perianal disease	3/15	4/15	0.805
Baseline HBI	6 [4 to 8]	9 [8 to 9.5]	0.014
Baseline SES-CD score	6 [4 to 8]	13 [6 to 18]	0.019
Baseline imaging **			
Inflammation	10/15	14/15	0.44
Stenosis	0/15	10/15	<0.001
Fistulae	0/15	4/15	0.079
Abscess (intraabdominal)	0/15	2/15	0.303
Baseline medication			
5-ASA	10/15	6/15	0.136
Azathioprine	9/15	11/15	0.35
Budesonide/Prednisolone	4/15	2/15	0.326
Anti-TNFα#	8/15	14/15	0.018
History of IBD-related surgery	15	15	0.086

Results are expressed as median [Q1 to Q3], where Q is the value of quartile; {min to max} for quantitative data and frequencies for qualitative data. B1 = non-stricturing, non-penetrating; B2 = stricturing; B3 = penetrating; HBI = Harvey–Bradshaw index; # Infliximab/Adalimumab. * Mann–Whitney test for quantitative data/Fisher’s exact test for qualitative data; ** CT/MRI/Intestinal US.

**Table 2:** Summary of clinical outcomes at 1-year follow up.

Characteristic at 1-Year Follow Up	Crohn’s Disease B1 (n = 15)	Crohn’s Disease B2B3 (n = 15)	*p*-Value *
HBI	2 [2 to 3] {0 to 6}	3 [3 to 4.5] {0 to 11}	0.1533
CRP (mg/dL)	0.44 [0.35 to 0.5] {0.28 to 2.56}	0.43 [0.335 to 0.975] {0.28 to 18.54}	0.9449
ALB (g/dL)	4.1 [3.9 to 4.2] {3.6 to 5.2}	4.2 [3.85 to 4.45] {3.6 to 5.3}	0.7471
FCal (µg/g)	50 [50 to 65] {5 to 240}	70 [50 to 141] {18 to 700}	0.2136
SES-CD score	2 [1.5 to 4.25]{0 to 5}	5 [3 to 6]{0 to 8}	0.1779
Surgery	1/13	5/15	0.2338
Treatment escalation/Biologic change	3/13	2/15	0.7345

Results are expressed as median [Q1 to Q3], where Q is the value of quartile; {min to max} for quantitative data and report for qualitative data. * Mann–Whitney test was used to compare quantitative data, while Fisher’s exact test was applied for qualitative data. B1 = non-stricturing, non-penetrating; B2 = stricturing; B3 = penetrating; HBI = Harvey–Bradshaw index; SES-CD Score = Simple Endoscopic CD Score.

**Table 3:** Serum proteome alterations in the stricturing and penetrating Crohn’s disease phenotypes and healthy controls.

No.	Protein Name	Gene	ANOVA, x = Significant			Fold Change			
			B1 vs. HC	B2B3 vs. HC	B2B3 vs. B1	B1 vs. HC	B2B3 vs. HC	B2B3 vs. B1	B1 vs. B2B3
1	Albumin	*ALB*		x	x	0.68	0.19	0.28	**3.54**
2	Alpha-1-antichymotrypsin	*SERPINA3*		x	x	1.35	**1.77**	1.31	0.76
3	Charged multivesicular body protein 3	*CHMP3*		x	x	0.63	0.24	0.38	**2.61**
4	Complement component C9	*C9*		x		1.40	**1.59**	1.14	0.88
5	Complement factor I	*CFI*		x		1.13	1.34	1.18	0.85
6	DNA polymerase epsilon catalytic subunit A	*POLE*		x	x	1.28	**1.52**	1.19	0.84
7	Dynein axonemal heavy chain 7	*DNAH7*	x	x		0.70	0.26	0.37	2.69
8	Epididymal secretory glutathione peroxidase	*GPX5*	x	x		0.58	0.11	0.20	5.07
9	GDH/6PGL endoplasmic bifunctional protein	*H6PD*		x	x	1.61	2.33	1.45	0.69
10	Haptoglobin	*HP*	x	x		**7.95**	**7.72**	0.97	1.03
11	Haptoglobin-related protein	*HPR*		x	x	2.20	**2.92**	1.33	0.75
12	High mobility group nucleosome binding domain 5	*HMGN5*	x	x		0.89	1.36	1.53	0.65
13	Immunoglobulin kappa constant	*IGKC*	x	x		1.26	**1.70**	1.35	0.74
14	Leucine-rich alpha-2-glycoprotein	*LRG1*		x	x	1.46	**2.33**	**1.60**	0.63
15	Lumican	*LUM*	x	x		0.71	0.39	0.55	1.83
16	Lysine-specific demethylase 3A	*KDM3A*		x	x	0.69	0.31	0.45	**2.24**
17	Microtubule-associated protein 1A	*MAP1A*		x	x	1.43	1.48	1.04	0.97
18	Phosphatidylinositol 4,5-bisphosphate 3-kinase catalytic γ	*PIK3CG*		x	x	0.64	0.18	0.28	**3.58**
19	Phosphatidylinositol 5-phosphate 4-kinase type-2 α	*PIP4K2A*		x		1.13	1.47	1.30	0.77
20	Plasma serine protease inhibitor	*SERPINA5*		x	x	0.68	0.33	0.48	**2.07**
21	Plexin-A2	*PLXNA2*		x	x	0.66	0.22	0.33	**2.99**
22	PTB domain-containing engulfment adapter protein 1	*GULP1*		x	x	1.21	**1.54**	1.27	0.79
23	Putative inactive neutral ceramidase B	*ASAH2B*		x		1.36	**1.86**	1.37	0.73
24	Serine/threonine-protein kinase OSR1	*OXSR1*		x		1.66	**1.56**	0.94	1.07
25	Serum amyloid A-1 protein	*SAA1*		x	x	2.56	**4.15**	**1.62**	0.62
26	Tetratricopeptide repeat protein 9A	*TTC9*	x	x		0.62	0.15	0.25	4.05
27	Transgelin-2	*TAGLN2*	x	x		1.39	1.46	1.05	0.95
28	Translationally controlled tumor protein	*TPT1*	x	x		1.47	**2.05**	1.40	0.72
29	WD repeat-containing protein 31	*WDR31*		x	x	4.69	**12.87**	**2.74**	0.36

ANOVA with post hoc Tukey’s HSD = Tukey’s Honest Significant Difference (HSD) test, significant if *p* < 0.05; HC = healthy controls; B1 = non-stricturing, non-penetrating; B2 = stricturing; B3 = penetrating; fold change calculated as the ratio of the two group means, with bold values > 1.5.

**Table 4:** Correlation of potential biomarkers with diagnosis and clinical biomarkers.

No.	Protein Name	Gene	Diagnosis	HBI	CRP	ALB	FCal	HBI at 1 Year
1	WD repeat-containing protein 31	*WDR31*	0.42	0.50	**0.65**	−0.32	0.39	0.03
2	Leucine-rich alpha-2-glycoprotein	*LRG1*	0.45	0.40	0.48	−0.15	0.37	0.04
3	Serum amyloid A-1 protein	*SAA1*	0.50	0.40	**0.61**	−0.28	0.37	0.06
4	Phosphatidylinositol 4,5-bisphosphate 3-kinase catalytic subunit gamma isoform	*PIK3CG*	−0.54	−0.51	−0.20	−0.03	−0.23	−0.14
5	Albumin	*ALB*	−0.57	−0.53	−0.30	0.13	−0.25	−0.14
6	Plexin-A2	*PLXNA2*	−0.53	−0.57	−0.29	0.14	−0.30	−0.15
7	Charged multivesicular body protein 3	*CHMP3*	−0.48	−0.44	−0.37	−0.01	−0.19	−0.09
8	Lysine-specific demethylase 3A	*KDM3A*	−0.53	−0.45	−0.22	0.14	−0.17	−0.14
9	Plasma serine protease inhibitor	*SERPINA5*	−0.42	−0.49	−0.42	0.16	−0.23	−0.02

HBI = Harvey–Bradshaw index; CRP = C-reactive protein; ALB = albumin; FCal = fecal calprotectin, with bold values > 0.6.

## Data Availability

Data is contained within the article and supplementary material. Any additional data can be obtained by request from the corresponding author.

## Supplementary Materials

The following supporting information can be downloaded at: https://www.mdpi.com/article/10.3390/ijms242316966/s1.
