# Supplementary material for: Unveiling Distinct Proteomic Signatures in Complicated Crohn’s Disease That Could Predict the Disease Course
Source: Int J Mol Sci. 2023 Nov 30;24(23):16966. doi: 10.3390/ijms242316966 (PMC10707401; doi:10.3390/ijms242316966)
Supplement: Supplementary file 1 [file ijms-24-16966-s001.zip › Suplementary Table S1. Overview on the abundance of all relatively quantified 770 proteins.pdf]

Supplementary Table S1. Overview on the abundance of all relatively quantified 770 proteins

			Group->	HC	HC	HC	HC	HC	HC	HC	HC	HC	HC	HC	HC	HC	HC	HC	B1	B1	B1	B1	B1	B1	B1	B1	B1	B1	B1	B1	B1	B1	B1	B2B3	B2B3	B2B3	B2B3	B2B3	B2B3	B2B3	B2B3	B2B3	B2B3	B2B3	B2B3	B2B3	B2B3	B2B3
No.	Protein name	UniProtID	Gene	P01	P04	P07	P10	P13	P16	P19	P22	P25	P28	P31	P34	P37	P40	P43	P02	P05	P08	P11	P14	P17	P20	P23	P26	P29	P32	P35	P38	P41	P44	P03	P06	P09	P12	P15	P18	P21	P24	P27	P30	P33	P36	P39	P42	P45
1	14-3-3 protein eta	Q04917	YWHAH	5960	6150	9360	7860	7630	6680	7480	3010	6310	4510	5230	4810	5850	6780	12300	4220	6620	10200	10100	10000	6920	6610	5730	5010	9180	6180	8510	12600	7860	11600	8810	8550	7770	8910	5860	8550	5510	5320	5520	5590	8040	6500	12400	18900	11000
2	1-phosphatidylinositol 3-phosphate 5-kinase	Q9Y2I7	PIKFYVE	295	345	231	333	346	576	376	445	737	354	567	582	639	362	544	278	441	271	218	519	425	498	423	444	287	540	359	495	447	295	253	390	288	258	347	381	654	446	399	486	335	277	637	303	352
3	1-phosphatidylinositol 4_5-bisphosphate phosphodiesterase epsilon-1	Q9P212	PLCE1	11900	15500	12200	13400	15000	17600	15500	15400	22300	15000	16700	11900	21100	24700	28100	10500	9620	16400	15500	13500	13400	13900	14300	14000	13800	23500	21500	20900	23800	27800	10000	17900	19100	17600	14600	20800	17600	23800	14100	20400	18100	17300	18900	25700	28100
4	1-phosphatidylinositol 4_5-bisphosphate phosphodiesterase eta-2	O75038	PLCH2	4970	6190	1240	6170	3190	6070	4200	4630	3950	5210	5430	5140	1470	5010	8820	1260	2010	2120	806	6740	4090	6120	6110	3330	1300	1390	2330	7080	4310	7720	1280	3780	2520	1730	4480	3810	14500	2800	1460	3250	1680	5710	5770	1900	869
5	2-oxoglutarate and iron-dependent oxygenase domain-containing protein 2	Q6N063	OGFOD2	3380	8390	2010	4470	3530	7440	5370	6100	3090	5580	6140	5750	3070	6030	5370	2210	3140	2020	2310	8110	5550	2360	9080	3810	1690	2230	3660	4970	5250	8520	2630	5850	5380	3040	3820	4490	10200	2790	2920	4090	2820	6910	7720	3990	2470
6	40S ribosomal protein SA	P08865	RPSA	54000	93800	40800	43400	51600	54400	44100	59300	27900	60900	43100	49000	54700	76000	50900	70000	56900	41400	43000	33000	45600	51800	39300	41600	26000	41600	37900	34800	45500	61200	49700	46300	38600	60200	50000	38600	52000	47900	30200	37100	39700	71800	47300	37800	69300
7	60S ribosomal protein L5	P46777	RPL5	67	18	116	167	114	35	92	18	13	14	11	13	2		27	16	67	100	175	59	36	32	111	11	65	8	43	185	8	10	111	524	368	144	28	72	78	3	120	30	126	41	130	138	
8	Actin filament-associated protein 1-like 1	Q8TED9	AFAP1L1	969	510	671	927	826	378	720	649	916	905	924	919	873	810	1130	1140	924	729	795	878	614	931	1280	742	1270	985	1440	1070	729	1100	746	839	1800	659	653	700	1100	613	949	979	1260	989	852	1590	1240
9	Actin_ aortic smooth muscle	P62736	ACTA2	947	1650	1370	476	1020	799	859	596	723	185	676	626	586	1010	729	631	2220	2500	306	5810	838	3580	1900	218	135	301	685	16	1620	138	485	619	520	546	2310	658	668	1180	73	202	215	314	430	2010	943
10	Actin_ cytoplasmic 2	P63261	ACTG1	4250	3960	3450	4920	4990	3140	4860	3900	4280	4260	7240	5180	4910	2730	4860	2120	5110	4600	3320	11500	3710	12100	14500	2810	2380	2350	7660	5590	5920	4000	2760	4340	7820	2990	6010	3740	25300	3070	5870	3100	9640	8900	8580	15900	3210
11	Activating signal cointegrator 1 complex subunit 3	Q8N3C0	ASCC3	671	468	507	548	187	269	1080	1570	1760	2030	1770	2150	2490	2950	2870	540	593	399	194	464	731	1350	759	1830	1040	1330	1730	835	2550	2320	272	199	109	210	296	460	1180	983	889	1170	662	1800	2500	1110	1140
12	Activating transcription factor 7-interacting protein 1	Q6VMQ6	ATF7IP	3140	1330	3330	3650	3030	2860	3410	2610	2480	1770	2370	2820	2490	2470	3220	2880	2860	4810	3720	2870	2880	6370	3720	2360	2650	1820	3190	3380	3970	2500	3770	4240	5230	3190	2280	3540	6120	1940	2090	2510	3010	2330	3140	3050	2300
13	Acyl-coenzyme A thioesterase THEM4	Q5T1C6	THEM4	472	476	452	556	869	741	911	795	1230	991	1140	1290	739	572	968	317	357	660	637	665	601	1020	1450	1030	805	878	1350	1760	825	985	601	800	1450	636	573	853	2320	672	1190	1130	1420	1430	1730	1600	694
14	Acylpyruvase FAHD1_ mitochondrial	Q6P587	FAHD1	31300	6140	15600	17200	24000	26400	24800	45900	12100	16500	8760	44500	25300	111000	14500	66100	13300	13800	6090	13300	17800	15000	7040	29800	23500	21700	11300	7980	67500	13800	10000	7420	5930	3620	7000	11600	11700	25400	9970	14700	6030	6540	5110	12300	19300
15	Adenylate cyclase type 9	O60503	ADCY9	8710	5730	7270	10400	9110	5960	6480	5200	7080	5730	7860	7270	9460	6110	10500	5870	7190	7060	9080	10100	9540	7610	14200	5060	5810	6730	9200	10300	6780	9850	11500	5880	9530	12400	7650	9520	13900	8380	8720	5450	7820	13400	13600	17900	12400
16	Adenylate kinase 9	Q5TCS8	AK9	333	239	433	315	286	359	333	134	392	354	275	301	229	334	382	214	282	354	370	306	318	279	273	265	371	275	357	428	401	364	433	373	57	321	331	391	204	200	429	2460	361	379	509	570	253
17	Adiponectin	Q15848	ADIPOQ	1550	1220	727	928	930	1870	2160	2070	514	1380	3980	2570	1350	2040	1280	707	959	814	1020	1860	1100	2550	1790	610	411	1430	873	1010	1120	2160	844	1080	4520	2300	2120	848	2110	1410	2330	2670	1420	3620	1410	860	1450
18	ADP-ribosylation factor 1	P84077	ARF1	19	3	34	7	6		24				8	12	7			2		267	249	8	2	252	23	16	2410		111	4420		13	73	617	1270	597	6	472	141		488	174	937	8	132	10	75
19	ADP-ribosylation factor GTPase-activating protein 1	Q8N6T3	ARFGAP1	774	1340	1000	690	1150	1330	606	435	609	812	730	404	310	140	383	693	913	716	715	1150	459	451	1310	512	630	667	1000	309	143	287	1440	657	881	771	1050	403	933	741	1140	834	736	1360	551	847	235
20	ADP-ribosylation factor-like protein 8A	Q96BM9	ARL8A	60200	86300	89100	71300	83000	92400	83600	71200	51400	71300	72300	65300	96800	86900	119000	53400	60900	93600	106000	88400	80900	75900	91000	66900	96300	55400	101000	149000	130000	143000	65500	111000	85900	126000	88500	105000	129000	80500	133000	135000	122000	106000	114000	162000	132000
21	AF4/FMR2 family member 4	Q9UHB7	AFF4	6980	5200	3310	3700	4640	5000	3560	4560	5180	3050	5700	4380	5790	6700	5750	2480	3620	3700	3720	3360	4550	4370	2910	3210	4390	3980	6990	6010	5620	6640	4070	2520	8980	3660	4240	4260	7250	5050	4030	62700	3670	5320	4190	5940	6040
22	Afamin	P43652	AFM	262000	285000	247000	258000	213000	220000	201000	234000	251000	207000	235000	228000	333000	382000	396000	225000	300000	198000	171000	195000	236000	277000	286000	217000	146000	261000	294000	220000	344000	288000	183000	228000	235000	157000	216000	216000	352000	285000	164000	255000	132000	345000	294000	359000	327000
23	A-kinase anchor protein 12	Q02952	AKAP12	99500	187000	112000	117000	192000	210000	119000	140000	97400	124000	160000	146000	205000	240000	249000	111000	115000	127000	122000	140000	157000	168000	156000	118000	109000	163000	162000	153000	223000	259000	124000	105000	106000	152000	159000	110000	138000	214000	72900	129000	94400	241000	227000	268000	350000
24	A-kinase anchor protein 13	Q12802	AKAP13	42100	51100	39800	40000	44300	51400	44800	35500	41400	42700	47100	42000	45900	47400	59400	34600	45600	44100	41100	39500	52300	38000	40100	36500	43700	50500	46600	55400	53800	71900	42800	42900	45100	46100	38900	42200	35600	52700	43800	85700	45600	47700	52800	67400	75300
25	Albumin	P02768	ALB	3740000	398000	1880000	2350000	3330000	3260000	3290000	6710000	335000	2820000	636000	7530000	7190000	18800000	2500000	7350000	1330000	983000	521000	425000	1840000	1170000	1180000	5220000	3650000	1960000	1290000	884000	13600000	2430000	961000	294000	347000	232000	454000	980000	374000	2330000	679000	751000	355000	626000	619000	2300000	1090000
26	Alpha-1_6-mannosylglycoprotein 6-beta-N-acetylglucosaminyltransferase A	Q09328	MGAT5	3680	5510	3780	4270	5070	4290	3680	3580	1950	2940	4110	3060	2960	2350	4100	2300	3070	3470	4840	3660	3670	2710	6470	2780	3140	3030	3340	5030	2760	4160	3260	6090	7800	5070	3760	3420	5970	4450	3550	2610	6150	4000	4640	6190	2990
27	Alpha-1-acid glycoprotein 1	P02763	ORM1	660000	823000	963000	646000	729000	751000	944000	646000	379000	654000	789000	588000	1040000	784000	1180000	562000	537000	967000	1110000	881000	739000	856000	777000	532000	751000	494000	1110000	1850000	1270000	1620000	896000	896000	689000	1270000	926000	1080000	1060000	694000	1440000	1430000	1320000	1060000	1180000	1940000	1580000

Supplementary Table S1. Overview on the abundance of all relatively quantified 770 proteins

No.	Protein name	UniProtID	Gene	HC P01	HC P04	HC P07	HC P10	HC P13	HC P16
268	GDH/6PGL endoplasmic bifunctional protein	O95479	H6PD	1650	480	2360	1840	1460	1740
269	Gelsolin	P06396	GSN	458000	369000	306000	373000	442000	366000
270	Girdin	Q3V6T2	CCDC88A	33600	36600	33100	42500	37200	39700
271	Glucose-6-phosphate 1-dehydrogenase	P11413	G6PD	3890	3890	2480	3220	2990	4480
272	Glucosylceramide transporter ABCA12	Q86UK0	ABCA12	11900	19900	8340	11000	10500	15500
273	Glutamate-rich protein 5	Q6P6B1	ERICH5	9130	10600	6670	8780	9460	9540
274	Glutaredoxin domain-containing cysteine-rich protein 2	A6NFK2	GRXCR2	95700	156000	67900	96600	121000	134000
275	Glutathione hydrolase 1 proenzyme	P19440	GGT1	6640	3340	3070	6380	4190	2660
276	Glutathione peroxidase 3	P22352	GPX3	34100	32700	35500	33300	28600	22700
277	Glutathione S-transferase theta-4	A0A1W2PR19	GSTT4	492	819	700	402	227	882
278	Glyceraldehyde-3-phosphate dehydrogenase	P04406	GAPDH	2770	3030	2400	2420	4370	2840
279	Glycerol-3-phosphate dehydrogenase 1-like protein	Q8N335	GPD1L	32000	24700	18700	23800	37100	39000
280	Glycine N-acyltransferase-like protein 1	Q969I3	GLYATL1	16700	24300	16700	16700	16700	12900
281	Glycogen phosphorylase_ muscle form	P11217	PYGM	12000	12900	16100	11600	17900	16700
282	Glycosyl-phosphatidylinositol-anchored molecule-like protein	Q99445	GML	3580	4190	5250	3500	5670	5540
283	Golgi phosphoprotein 3	Q9H4A6	GOLPH3	2950	2930	3000	2720	3190	4060
284	Golgin subfamily A member 3	Q08378	GOLGA3	1280	2430	1420	1780	2290	2450
285	Golgin subfamily A member 4	Q13439	GOLGA4	2500	3370	3310	3150	3120	3190
286	Golgin subfamily B member 1	Q14789	GOLGB1	27600	33800	24000	23100	26800	32000
287	Grainyhead-like protein 2 homolog	Q6ISB3	GRHL2	2870	5600	3570	2850	2510	3900
288	GRB2-related adapter protein	Q13588	GRAP	14100	5890	8660	11500	16100	13700
289	GRIP and coiled-coil domain-containing protein 2	Q8IWJ2	GCC2	5160	6690	7580	5910	5890	7520
290	Guanine nucleotide exchange factor DBS	O15068	MCF2L	2170	1220	717	1000	993	1740
291	Guanine nucleotide exchange factor MSS4	P47224	RABIF	2350	2530	1830	2310	3330	3500
292	Guanine nucleotide-binding protein-like 3-like protein	Q9NVN8	GNL3L	5360	6940	8870	6150	6890	7920
293	Guanylate-binding protein 2	P32456	GBP2	165	247	464	112	96	172
294	Haptoglobin	P00738	HP	54400	26900	47400	34000	31500	21700
295	Haptoglobin-related protein	P00739	HPR	10600	5330	13300	7660	6780	6990
296	Heat shock 70 kDa protein 4L	O95757	HSPA4L	2360	2280	1220	1370	1420	2050
297	Heat shock protein HSP 90-alpha	Q14568	HSP90AA2P	1520	3940	993	1360	1490	2500
298	Heat shock protein HSP 90-alpha A2	P07900	HSP90AA1	2090	3480	2860	2340	2940	3580
299	Helicase SRCAP	Q6ZRS2	SRCAP	9160	20500	9350	8290	10500	14600
300	Hematopoietically-expressed homeobox protein HHEX	Q03014	HHEX	681	1090	448	538	985	359
301	Hemicentin-2	Q8NDA2	HMCN2	958	314	1130	826	572	489
302	Hemoglobin subunit alpha	P69905	HBA1;	26800	34100	22600	22300	27100	23700
303	Hemoglobin subunit beta	P68871	HBB	34700	29900	28900	40300	50800	39400
304	Hemoglobin subunit delta	P02042	HBD	12000	17600	15700	11400	13800	13100
305	Hemoglobin subunit epsilon	P02100	HBE1	2550	3310	4030	3390	3940	3000
306	Hemoglobin subunit gamma-2	P69892	HBG2	36600	41400	37200	37500	45800	45200
307	Hemopexin	P02790	HPX	2130000	2360000	2140000	2070000	2240000	2230000
308	Heparin cofactor 2	P05546	SERPIND1	192000	164000	193000	176000	138000	183000
309	Hepatocyte growth factor activator	Q04756	HGFAC	31500	38800	37300	30200	30700	38600
310	Hepatocyte growth factor-like protein	P26927	MST1	59700	34200	66800	53200	54500	59000
311	Hepatocyte nuclear factor 3-beta	Q9Y261	FOXA2	1030	1170	1230	1820	1880	1110
312	Hepatocyte nuclear factor 3-gamma	P55318	FOXA3	1760	1800	1770	1750	1860	1680
313	HIG1 domain family member 1C	A8MV81	HIGD1C	89	175	161	90	113	
314	High mobility group nucleosome-binding domain-containing protein 5	P82970	HMGN5	5420	7720	5020	4690	6470	6110
315	Histidine protein methyltransferase 1 homolog	O95568	METTL18	3670	5880	4030	4100	4320	5110
316	Histidine-rich glycoprotein	P04196	HRG	310000	196000	189000	158000	228000	259000
317	Histidine--tRNA ligase_ cytoplasmic	P12081	HARS1	21700	24000	22100	23300	26200	21500
318	Histone acetyltransferase KAT7	O95251	KAT7	3780	3900	5080	3870	3990	5110
319	Histone-lysine N-methyltransferase 2A	Q03164	KMT2A	3150	3330	2410	3560	4430	2970
320	Histone-lysine N-methyltransferase PRDM16	Q9HAZ2	PRDM16	794	2050	610	422	656	1010
321	HIV Tat-specific factor 1	O43719	HTATSF1	8430	12500	9040	8430	9850	11500
322	HLA class II histocompatibility antigen_ DM beta chain	P28068	HLA-DMB	2250	1760	2300	1940	758	7
323	HLA class II histocompatibility antigen_ DO alpha chain	P06340	HLA-DOA	2360	5450	4080	1340	445	3660
324	HLA class II histocompatibility antigen_ DQ beta 2 chain	P05538	HLA-DQB2	31000	48800	40700	38800	40400	46500
325	Homeobox protein cut-like 2	O14529	CUX2	35500	63300	35900	36900	37300	43900
326	Homeobox protein HMX3	A6NHT5	HMX3	35500	46200	27700	24100	36300	28300
327	Homeobox protein Hox-C10	Q9NYD6	HOXC10	291	435	444	331	358	218
328	Homeobox protein Hox-C6	P09630	HOXC6	639	1200	992	415	820	866
329	Homeodomain-only protein	Q9BPY8	HOPX	1280	1270	1660	1490	1060	2040
330	Hyaluronan-binding protein 2	Q14520	HABP2	58400	72600	61300	60600	67200	78400
331	Hydroxyacyl-coenzyme A dehydrogenase_ mitochondrial	Q16836	HADH	1280	270	854	1130	1260	604
332	Immunoglobulin heavy constant alpha 1	P01876	IGHA1	52100	68100	57200	56000	66000	66000
333	Immunoglobulin heavy constant alpha 2	P01877	IGHA2	5530	6540	5560	6440	4630	4920
334	Immunoglobulin heavy constant delta	P01880	IGHD	13900	13200	10900	13900	14400	11200
335	Immunoglobulin heavy constant gamma 1	P01857	IGHG1	208	588	298	351	476	614
336	Immunoglobulin heavy constant gamma 2	P01859	IGHG2	17000	24200	9710	14700	18400	15200
337	Immunoglobulin heavy constant gamma 3	P01860	IGHG3	7170	3030	4410	6200	10700	11000
338	Immunoglobulin heavy constant mu	P01871	IGHM	35700	51100	98700	50700	81600	103000
339	Immunoglobulin heavy variable 1-58	A0A0C4DH39	IGHV1-58	176	252	281	231	117	63
340	Immunoglobulin heavy variable 3-7	P01780	IGHV3-7	828	1110	1350	812	1280	958
341	Immunoglobulin J chain	P01591	JCHAIN	119	208	985	380	809	997
342	Immunoglobulin kappa constant	P01834	IGKC	43600	45000	53500	38300	61800	49100
343	Immunoglobulin lambda constant 2	P0DOY2	IGLC2	556	60	1100	483	722	724
344	Immunoglobulin lambda constant 6	P0CF74	IGLC6	167	174	269	126	154	204
345	Immunoglobulin lambda variable 1-36	A0A0B4J1U3	IGLV1-36	1010	107	1020	568	476	711
346	Immunoglobulin lambda variable 3-12	A0A075B6K2	IGLV3-12	17100	47500	12700	13100	17500	27500
347	Inactive dipeptidyl peptidase 10	Q8N608	DPP10	1090	1760	2040	1780	1440	1840
348	Inactive tyrosine-protein kinase PEAK1	Q9H792	PEAK1	7050	8320	10000	5450	5650	9640
349	Induced myeloid leukemia cell differentiation protein Mcl-1	Q07820	MCL1	3100	2270	1430	1610	2290	1950
350	Inositol hexakisphosphate kinase 3	Q96PC2	IP6K3	2900	3930	3550	3580	3740	3580
351	Insulin-like growth factor II	P01344	IGF2	4190	6370	4060	3980	4750	6230
352	Insulin-like growth factor-binding protein 3	P17936	IGFBP3	11200	16200	13200	15000	17900	17000
353	Insulin-like growth factor-binding protein complex acid labile subunit	P35858	IGFALS	71300	81600	76400	76900	74600	95700
354	Integrator complex subunit 11	Q5TA45	INTS11	32300	48200	23600	30600	34700	34500
355	Integrator complex subunit 6	Q9UL03	INTS6	591	577	389	363	309	716
356	Integrator complex subunit 6-like	Q5JSJ4	INTS6L	13900	22700	16200	14900	7880	3730
357	Inter-alpha-trypsin inhibitor heavy chain H1	P19827	ITIH1	528000	572000	706000	353000	332000	458000
358	Inter-alpha-trypsin inhibitor heavy chain H2	P19823	ITIH2	796000	809000	933000	576000	645000	686000
359	Inter-alpha-trypsin inhibitor heavy chain H3	Q06033	ITIH3	103000	120000	123000	85200	132000	111000
360	Inter-alpha-trypsin inhibitor heavy chain H4	Q14624	ITIH4	702000	793000	863000	655000	695000	626000
361	Intercellular adhesion molecule 2	P13598	ICAM2	711	1190	925	861	785	930
362	Interferon-related developmental regulator 2	Q12894	IFRD2	17300	18400	17600	19600	23600	19600
363	Interleukin-18	Q14116	IL18	26200	3670	14900	19400	31100	37500
364	Interleukin-36 receptor antagonist protein	Q9UBH0	IL36RN	4110	6720	7650	4440	6120	4470
365	Intestine-specific homeobox	Q2M1V0	ISX	19000	13600	7070	17100	12600	13600
366	Intraflagellar transport protein 172 homolog	Q9UG01	IFT172	7060	16800	6050	6820	10600	9900
367	IQCJ-SCHIP1 readthrough transcript protein	B3KU38	IQCJ-SCHIP1	110000	104000	87400	98300	110000	90400
368	Jun dimerization protein 2	Q8WYK2	JDP2	78	10	157	243	70	
369	Kallistatin	P29622	SERPINA4	66500	69500	59400	65700	65900	57000
370	KAT8 regulatory NSL complex subunit 3	Q9P2N6	KANSL3	2930	1640	1390	1360	1210	1600
371	Katanin p60 ATPase-containing subunit A1	O75449	KATNA1	775	624	827	800	1010	747
372	Keratin_ type I cytoskeletal 10	P13645	KRT10	32900	35900	32800	28600	29200	31600
373	Keratin_ type I cytoskeletal 14	P02533	KRT14	3880	3700	2690	3070	3760	4500
374	Keratin_ type I cytoskeletal 15	P19012	KRT15	3300	5160	3220	3440	4440	4000
375	Keratin_ type I cytoskeletal 16	P08779	KRT16	176	377	378	276	316	436
376	Keratin_ type I cytoskeletal 17	Q04695	KRT17	619	263	742	242	241	485
377	Keratin_ type I cytoskeletal 20	P35900	KRT20	2210	1200	1570	2160	3010	3690
378	Keratin_ type I cytoskeletal 28	Q7Z3Y7	KRT28	3510	3840	3100	3720	4420	3210
379	Keratin_ type I cytoskeletal 9	P35527	KRT9	17000	26700	16500	17900	37700	15700
380	Keratin_ type II cytoskeletal 1	P04264	KRT1	43600	53200	40100	36200	45600	38000
381	Keratin_ type II cytoskeletal 1b	Q7Z794	KRT77	33800	52000	38500	34900	40900	40300
382	Keratin_ type II cytoskeletal 2 epidermal	P35908	KRT2	37700	108000	59900	47000	38200	37500
383	Keratin_ type II cytoskeletal 2 oral	Q01546	KRT76	3810	4890	3040	3230	5310	5120
384	Keratin_ type II cytoskeletal 4	P19013	KRT4	3340	4580	3520	3810	4230	4130
385	Keratin_ type II cytoskeletal 5	P13647	KRT5	2750	2970	2340	2150	2810	3090
386	Keratin_ type II cytoskeletal 6A	P02538	KRT6A	49	11	18	20	15	6
387	Keratin_ type II cytoskeletal 6B	P04259	KRT6B	229	218	146	207	248	135
388	Keratin_ type II cytoskeletal 7	P08729	KRT7	6660	10000	8920	6970	8050	9970
389	Keratin_ type II cytoskeletal 8	P05787	KRT8	3280	4280	2500	3140	3610	3220
390	Keratin_ type II cytoskeletal 80	Q6KB66	KRT80	16100	21000	17900	17800	17400	18800
391	Kinesin heavy chain isoform 5C	O60282	KIF5C	46500	32000	36500	42600	43400	52700
392	Kinesin-like protein KIF21B	O75037	KIF21B	5080	5730	3340	4110	4090	3210
393	Kinesin-like protein KIF27	Q86VH2	KIF27	5420	7130	3780	4280	6330	5090
394	Kininogen-1	P01042	KNG1	365000	405000	283000	355000	349000	372000
395	Klotho	Q9UEF7	KL	19100	29500	23600	21200	25600	26300
396	Lactosylceramide alpha-2_3-sialyltransferase	Q9UNP4	ST3GAL5	11800	22900	15700	15100	18200	24900
397	Lactotransferrin	P02788	LTF	13000	17400	9430	13700	16400	17300
398	Laminin subunit alpha-2	P24043	LAMA2	1580	2290	3150	1730	2440	1880
399	Legumain	Q99538	LGMN	15900	28300	12300	15000	16300	23000
400	LEM domain-containing protein 1	Q68G75	LEMD1	331		509	190		
401	Lethal(3)malignant brain tumor-like protein 3	Q96JM7	L3MBTL3	816	490	695	865	560	372
402	Leucine-rich alpha-2-glycoprotein	P02750	LRG1	85200	105000	141000	86600	112000	104000
403	Leucine-rich repeat flightless-interacting protein 2	Q9Y608	LRRFIP2	15700	26800	11900	13200	20300	26300
404	LIM and calponin homology domains-containing protein 1	Q9UPQ0	LIMCH1	22900	25000	22400	21100	19600	26300
405	LIM/homeobox protein Lhx9	Q9NQ69	LHX9	4170	1120	4210	3440	3440	4540
406	LINE-1 type transposase domain-containing protein 1	Q5T7N2	L1TD1	1900	2180	1400	1470	1560	1730
407	Lipopolysaccharide-binding protein	P18428	LBP	52500	43800	56200	61800	56800	49100
408	Lipoyl synthase_ mitochondrial	O43766	LIAS	4350	6600	3070	4310	4840	5500
409	L-lactate dehydrogenase A chain	P00338	LDHA	166	34	247	85	6	
410	Long-chain-fatty-acid--CoA ligase 5	Q9ULC5	ACSL5	13400	23400	12600	14900	22400	25100
411	Lumican	P51884	LUM	192000	85000	127000	143000	206000	224000
412	Lysine-specific demethylase 3A	Q9Y4C1	KDM3A	200000	35900	93100	131000	205000	163000
413	Lysine-specific demethylase 3B	Q7LBC6	KDM3B	757	1340	1210	667	1230	1830
414	Lysine-specific demethylase hairless	O43593	HR	713	733	801	665	764	666
415	Lysine-specific histone demethylase 1A	O60341	KDM1A	3440	3670	3760	3810	4260	3760
416	Lysophospholipid acyltransferase 2	Q6ZWT7	MBOAT2	1540	452	1170	1100	681	262
417	Lysosomal-trafficking regulator	Q99698	LYST	4660	6660	3730	5210	6580	4930
418	Lysozyme C	P61626	LYZ	11400	12100	9550	9890	10700	11400
419	Mammalian ependymin-related protein 1	Q9UM22	EPDR1	2740	4300	4190	2750	2990	3330
420	Mannan-binding lectin serine protease 1	P48740	MASP1	13300	19400	15000	13300	14700	14800
421	MAP6 domain-containing protein 1	Q9H9H5	MAP6D1	18300	19800	14900	14500	16100	19100
422	MAP7 domain-containing protein 1	Q3KQU3	MAP7D1	46700	55800	38300	40800	53200	49700
423	MAP7 domain-containing protein 3	Q8IWC1	MAP7D3	29000	34400	21100	24600	33600	29600
424	Melanoma-associated antigen 12	P43365	MAGEA12	18200	24600	18600	16300	22200	27100
425	Melanoma-associated antigen B10	Q96LZ2	MAGEB10	716	1190	729	677	1050	975
426	Melanoma-associated antigen C3	Q8TD91	MAGEC3	6450	6380	4420	5030	9320	11200
427	Melanophilin	Q9BV36	MLPH	29900	42100	29100	27100	43600	47000
428	Melanotransferrin	P08582	MELTF	3150	5980	4610	4010	3750	4620
429	MICOS complex subunit MIC60	Q16891	IMMT	29700	27300	22500	30100	43000	26000
430	Microtubule-associated protein 1A	P78559	MAP1A	697	732	1300	809	811	887
431	Microtubule-associated protein 2	P11137	MAP2	20100	8310	11100	11800	19600	17100
432	Microtubule-associated serine/threonine-protein kinase 4	O15021	MAST4	1030	1470	1830	1530	1120	1330
433	Microtubule-associated tumor suppressor candidate 2	Q5JR59	MTUS2	4000	4880	4720	5240	5390	3590
434	Mid1-interacting protein 1	Q9NPA3	MID1IP1	3600	4650	2060	2910	3260	1820
435	Midasin	Q9NU22	MDN1	6710	5900	4880	8250	10400	8560
436	Mitochondrial peptide methionine sulfoxide reductase	Q9UJ68	MSRA	269	201	349	372	369	247
437	Mitochondrial ribonuclease P catalytic subunit	O15091	PRORP	17400	26600	18800	22800	20900	18700
438	Mitogen-activated protein kinase kinase kinase 1	Q13233	MAP3K1	24	344	100	62	104	274
439	Mitogen-activated protein kinase kinase kinase 15	Q6ZN16	MAP3K15	6290	6300	5010	7380	10000	7350
440	Mitogen-activated protein kinase kinase kinase 5	Q99683	MAP3K5	75	164	93	114	129	45
441	Monoacylglycerol lipase ABHD2	P08910	ABHD2	51400	51900	47100	47200	53600	46200
442	Monocyte differentiation antigen CD14	P08571	CD14	139000	260000	105000	146000	187000	205000
443	MORC family CW-type zinc finger protein 1	Q86VD1	MORC1	1200	985	1140	768	958	1230
444	M-phase inducer phosphatase 1	P30304	CDC25A	24800	48000	20600	20900	25500	33300
445	M-phase phosphoprotein 9	Q99550	MPHOSPH9	4940	7520	9960	6720	4140	5670
446	MTRF1L release factor glutamine methyltransferase	Q9Y5R4	HEMK1	67300	72300	67900	64600	68800	79500
447	Mucin-16	Q8WXI7	MUC16	33800	41100	38500	29400	30400	38500
448	Mucin-19	Q7Z5P9	MUC19	39700	37200	23100	34000	34500	40000
449	Mucolipin-2	Q8IZK6	MCOLN2	1090	7	605	785	1310	1290
450	Multivesicular body subunit 12A	Q96EY5	MVB12A	10600	10400	13700	13600	12100	13900
451	Myb/SANT-like DNA-binding domain-containing protein 3	Q96H12	MSANTD3	7740	8540	9730	11000	13500	9230
452	MYCBP-associated protein	Q8TBZ2	MYCBPAP	621	305	1010	1310	1090	
453	Myosin light chain 5	Q02045	MYL5	218000	166000	288000	259000	124000	100000
454	Myosin regulatory light chain 2_ skeletal muscle isoform	Q96A32	MYL11	14300	22500	9510	12100	14800	17300
455	Myosin regulatory light chain 2_ ventricular/cardiac muscle isoform	P10916	MYL2	2080	2510	3060	2580	2620	3450
456	Myosin-10	P35580	MYH10	1400	2600	1020	1300	1860	1810
457	Myosin-11	P35749	MYH11	24100	24500	32400	23500	25200	25300
458	Myosin-15	Q9Y2K3	MYH15	9260	13300	9510	8450	10800	12000
459	Myosin-6	P13533	MYH6	59400	59400	48900	54900	67200	92500
460	Myosin-7B	A7E2Y1	MYH7B	76800	119000	43400	68500	85800	88700
461	Myosin-9	P35579	MYH9	10500	13400	11800	8360	11300	13100
462	Myotubularin-related protein 8	Q96EF0	MTMR8	8960	12700	10700	10300	11300	14000
463	Myozenin-2	Q9NPC6	MYOZ2	5510	8040	2680	2920	5020	2300
464	N-acetylmuramoyl-L-alanine amidase	Q96PD5	PGLYRP2	300000	359000	322000	319000	403000	387000
465	N-acetylneuraminate lyase	Q9BXD5	NPL	13700	13900	12100	12300	6870	14200
466	NACHT domain- and WD repeat-containing protein 1	Q149M9	NWD1	3710	2480	2120	3350	1550	3750
467	N-acylglucosamine 2-epimerase	P51606	RENBP	24100	36600	31400	22400	27500	27800
468	NAD-dependent protein deacetylase sirtuin-6	Q8N6T7	SIRT6	17	5	27	82	19	5
469	NADH dehydrogenase [ubiquinone] 1 alpha subcomplex subunit 13	Q9P0J0	NDUFA13	1510	2750	1790	1630	1930	1620
470	Nck-associated protein 1	Q9Y2A7	NCKAP1	112	9	64	67	47	18
471	N-cym protein	P40205	MYCNOS	721	953	1220	743	910	1080
472	Nesprin-1	Q8NF91	SYNE1	22000	22900	22600	28200	27100	21600
473	Nesprin-2	Q8WXH0	SYNE2	129000	166000	147000	142000	165000	156000
474	Neurexin-1	Q9ULB1	NRXN1	1030	69	1200	1080	714	113
475	Neurofibromin	P21359	NF1	25400	26300	14700	13200	29000	12800
476	Neuromodulin	P17677	GAP43	902	432	783	451	265	494
477	Neuron navigator 1	Q8NEY1	NAV1	22800	27900	20600	26800	18100	31000
478	Neuronal calcium sensor 1	P62166	NCS1	46200	66500	63000	49200	45000	61800
479	Neuronal cell adhesion molecule	Q92823	NRCAM	212		7	50	249	51
480	NFX1-type zinc finger-containing protein 1	Q9P2E3	ZNFX1	3830	5470	4400	4320	5220	5940
481	Nicotinamide N-methyltransferase	P40261	NNMT	7530	11900	7470	7200	8200	11300
482	Nipped-B-like protein	Q6KC79	NIPBL	6400	9770	7900	5560	8500	7250
483	Noelin	Q99784	OLFM1	2090	2630	1980	1790	3200	2850
484	N-terminal EF-hand calcium-binding protein 2	Q7Z6G3	NECAB2	243	262	400	267	182	154
485	N-terminal Xaa-Pro-Lys N-methyltransferase 1	Q9BV86	NTMT1	42		17	36	11	
486	Nuclear cap-binding protein subunit 2-like	A6PVI3	NCBP2L	517	306	263	766	148	40
487	Nuclear mitotic apparatus protein 1	Q14980	NUMA1	8010	5280	3680	5730	8630	7460
488	Nuclear receptor subfamily 2 group C member 1	P13056	NR2C1	2650	3220	4690	6730	1920	4090
489	Nuclear receptor subfamily 2 group F member 6	P10588	NR2F6	667	42	427	1350	1880	104
490	Nuclear RNA export factor 5	Q9H1B4	NXF5	22100	30600	23100	18200	18400	29800
491	Nucleolysin TIAR	Q01085	TIAL1	8830	14100	7060	7310	9650	14300
492	Nucleoredoxin-like protein 1	Q96CM4	NXNL1	36500	41500	30500	30600	35400	41200
493	Obscurin	Q5VST9	OBSCN	14700	16100	17900	17200	19200	19900
494	Olfactory receptor 2B6	P58173	OR2B6	1860	1140	1800	2250	2070	1430
495	Oncomodulin-1	P0CE72	OCM	4020	2490	6390	5750	1460	246
496	Oral-facial-digital syndrome 1 protein	O75665	OFD1	430		23	33	71	52
497	Organic solute transporter subunit beta	Q86UW2	SLC51B	250	42	724	224		7
498	Oxysterol-binding protein 1	P22059	OSBP	180		406	247	10	
499	Oxysterol-binding protein 2	Q969R2	OSBP2	31000	31900	28100	26900	28700	31900
500	Oxysterol-binding protein-related protein 1	Q9BXW6	OSBPL1A	693	438	465	619	669	636
501	P antigen family member 1	O75459	PAGE1	346	277	450	255	132	338
502	p53 and DNA damage-regulated protein 1	Q9NUG6	PDRG1	2890	3480	3230	3680	3030	3260
503	Paired box protein Pax-8	Q06710	PAX8	50800	18200	56100	51500	28200	6370
504	Pantetheinase	O95497	VNN1	1460	63200	6240	1250	1440	1440
505	Pappalysin-1	Q13219	PAPPA	4970	4670	4790	5630	5360	4460
506	Parkinson disease protein 7	Q99497	PARK7	379	510	733	496	723	907
507	PAS domain-containing serine/threonine-protein kinase	Q96RG2	PASK	17300	7990	12500	14500	21700	21500
508	Paternally-expressed gene 3 protein	Q9GZU2	PEG3	7000	6560	4410	7250	9740	9670
509	PDZ domain-containing protein 11	Q5EBL8	PDZD11	23100	43300	37300	25700	37600	28600
510	Peptidyl-prolyl cis-trans isomerase C	P45877	PPIC	791	534	1130	1060	479	871
511	Peroxiredoxin-1	Q06830	PRDX1	17600	18700	30100	24300	13100	8400
512	Peroxiredoxin-2	P32119	PRDX2	13700	20400	12500	13200	21000	16000
513	Pescadillo homolog	O00541	PES1	7250	6030	6140	6120	6980	6050
514	Phosphatidylcholine-sterol acyltransferase	P04180	LCAT	15400	9450	10600	13700	12600	14100
515	Phosphatidylinositol 4_5-bisphosphate 3-kinase catalytic subunit alpha isoform	P42336	PIK3CA	192	259	325	63	115	226
516	Phosphatidylinositol 4_5-bisphosphate 3-kinase catalytic subunit gamma isoform	P48736	PIK3CG	33800	5530	17200	21800	32700	32700
517	[illegible]	[illegible]	[illegible]	[illegible]	[illegible]	[illegible]	[illegible]	[illegible]	[illegible]
518	[illegible]	[illegible]	[illegible]	[illegible]	[illegible]	[illegible]	[illegible]	[illegible]	[illegible]
519	[illegible]	[illegible]	[illegible]	[illegible]	[illegible]	[illegible]	[illegible]	[illegible]	[illegible]
520	[illegible]	[illegible]	[illegible]	[illegible]	[illegible]	[illegible]	[illegible]	[illegible]	[illegible]
521	[illegible]	[illegible]	[illegible]	[illegible]	[illegible]	[illegible]	[illegible]	[illegible]	[illegible]
522	[illegible]	[illegible]	[illegible]	[illegible]	[illegible]	[illegible]	[illegible]	[illegible]	[illegible]
523	[illegible]	[illegible]	[illegible]	[illegible]	[illegible]	[illegible]	[illegible]	[illegible]	[illegible]
524	[illegible]	[illegible]	[illegible]	[illegible]	[illegible]	[illegible]	[illegible]	[illegible]	[illegible]
525	[illegible]	[illegible]	[illegible]	[illegible]	[illegible]	[illegible]	[illegible]	[illegible]	[illegible]
526	[illegible]	[illegible]	[illegible]	[illegible]	[illegible]	[illegible]	[illegible]	[illegible]	[illegible]
527	[illegible]	[illegible]	[illegible]	[illegible]	[illegible]	[illegible]	[illegible]	[illegible]	[illegible]
528	[illegible]	[illegible]	[illegible]	[illegible]	[illegible]	[illegible]	[illegible]	[illegible]	[illegible]
529	[illegible]	[illegible]	[illegible]	[illegible]	[illegible]	[illegible]	[illegible]	[illegible]	[illegible]
530	[illegible]	[illegible]	[illegible]	[illegible]	[illegible]	[illegible]	[illegible]	[illegible]	[illegible]
531	[illegible]	[illegible]	[illegible]	[illegible]	[illegible]	[illegible]	[illegible]	[illegible]	[illegible]
532	[illegible]	[illegible]	[illegible]	[illegible]	[illegible]	[illegible]	[illegible]	[illegible]	[illegible]
533	[illegible]	[illegible]	[illegible]	[illegible]	[illegible]	[illegible]	[illegible]	[illegible]	[illegible]
534	[illegible]	[illegible]	[illegible]	[illegible]	[illegible]	[illegible]	[illegible]	[illegible]	[illegible]

No.	HC P19	HC P22	HC P25	HC P28	HC P31	HC P34	HC P37	HC P40	HC P43	B1 P02	B1 P05	B1 P08	B1 P11	B1 P14	B1 P17	B1 P20	B1 P23	B1 P26	B1 P29	B1 P32	B1 P35	B1 P38	B1 P41	B1 P44	B2B3 P03	B2B3 P06	B2B3 P09	B2B3 P12	B2B3 P15	B2B3 P18	B2B3 P21	B2B3 P24	B2B3 P27	B2B3 P30	B2B3 P33	B2B3 P36	B2B3 P39	B2B3 P42	B2B3 P45
268	1610	1550	3710	1090	1410	1420	1160	1530	1380	1190	1610	2700	2900	1660	2210	2610	2500	1460	3800	2200	1320	7300	2670	3090	3750	3910	7230	4470	2560	2330	3010	2150	4550	1730	7710	1540	2780	3140	5930
269	345000	409000	176000	319000	410000	318000	372000	468000	606000	353000	346000	273000	254000	278000	326000	343000	345000	253000	175000	225000	313000	310000	513000	491000	286000	268000	207000	241000	299000	263000	326000	451000	123000	169000	150000	405000	569000	473000	260000
270	26300	36400	24200	28300	42100	32800	33200	31500	45900	22500	31100	36300	48600	34500	39800	43900	50600	27200	65600	25800	48100	93600	40600	56600	26200	43500	52400	52500	30400	47800	69200	51900	44200	52000	61400	61200	60900	73000	51700
271	3390	3610	2610	4030	4010	5340	6500	6920	6930	3060	4150	3370	1660	2890	6070	4900	2080	3320	4240	6000	7000	1560	5840	4380	1670	1400	2440	1190	2830	2620	2550	6830	1180	6580	1050	4060	3340	3370	4030
272	10900	13100	9140	14600	11700	11800	10700	13000	12600	12500	11000	7850	9420	9780	11300	13200	9900	11000	4600	9450	8470	10100	9540	13900	9250	10500	33100	12800	12300	8480	11000	11700	9280	8430	12300	14200	9930	8350	11300
273	9650	11300	3300	6780	8950	7780	10300	10300	10000	10100	9720	7930	7720	10400	8320	7880	8630	5280	9930	7280	8460	9720	14400	13500	9090	7390	13200	11300	8500	9620	11500	9420	10000	8850	7990	9490	6360	15100	9290
274	104000	136000	85500	119000	133000	115000	133000	149000	160000	101000	89300	75000	101000	73900	92400	117000	108000	96900	41500	112000	94400	139000	134000	203000	87800	79200	96200	138000	121000	79100	116000	101000	90500	93100	103000	180000	153000	114000	192000
275	3440	3920	2570	3160	3570	3030	2200	2010	5060	10000	9030	4800	3990	4990	1850	4310	3580	2360	5250	3920	4490	1930	1620	3600	4670	3120	1010	1590	2960	2300	3480	3850	1640	2500	2080	1830	374	3070	2510
276	36200	35400	33400	38800	41000	43300	46200	46700	56000	32100	33500	28800	37200	24600	30900	37800	36900	34900	35300	34100	43400	50700	44200	54400	35400	36500	33800	33200	24600	34800	38500	33900	39400	29200	47700	57400	55000	52500	50000
277	592	737	1160	613	481	272	736	306	725	621	395	1480	433	740	853	335	259	452	1010	684	628	883	681	1880	622	415	304	496	639	893	227	1330	497	1860	1290	693	470	364	1960
278	2390	2700	2360	2010	3210	2220	2420	4710	4500	1520	2670	3140	2270	4660	4070	4950	3180	1760	3020	3630	4220	3480	5270	2810	2620	1570	4280	2480	3450	2460	2490	6490	1940	1780	3900	2370	3150	2730	2360
279	29300	60500	15800	36600	24200	68900	60600	157000	35100	44100	20600	15200	18100	14600	24200	23900	19900	49500	29600	25900	20800	25000	109000	41800	17500	14400	21300	21900	20500	18600	25300	27900	20100	33700	23700	33600	27400	29900	32000
280	17000	22500	14000	22700	19000	24300	22400	27100	24300	17800	19600	15800	15900	14000	13800	14300	16700	17300	18800	18900	19300	23200	21700	23100	19400	15700	13900	16900	16200	16500	15100	17500	16000	15600	20400	25400	21600	23600	23500
281	12600	8680	15400	9230	12800	17800	27400	28700	27300	8050	9470	20600	19900	15200	11700	16800	12400	9710	17900	19000	25300	25900	36800	31500	17000	12800	11700	19000	14900	20900	14900	19100	17200	25000	18800	16000	22300	31500	25300
282	4450	2950	4330	2390	3190	4380	7320	7410	9370	2700	2590	6930	6560	4800	6290	6120	3820	3260	4940	4560	8700	10500	11700	13000	5350	3780	12900	6170	4770	7160	4450	5440	6160	5230	6910	5720	8320	9710	8950
283	2780	2450	2940	2870	2610	2950	4090	5090	5720	2300	3010	3110	3200	3040	3440	2970	2880	2640	1530	3410	3920	4880	5360	5440	3100	2860	2230	3120	3550	2910	2060	4360	2860	2960	2930	4360	5690	6470	5770
284	1940	1850	1410	1270	1990	1950	2130	2530	3300	1440	1640	2040	1970	2350	1940	2720	1670	1370	1580	1600	1860	2320	1750	2830	1730	1300	2120	3280	1890	2460	1250	3340	1480	1870	2390	1950	2600	2550	3120
285	3150	4090	3310	3520	3040	4980	4220	7940	4940	2100	2930	2960	3350	2850	3400	3130	3460	4030	4350	3640	3670	4180	6290	3500	3080	3890	3990	3240	2980	3000	3610	3310	3890	3130	3340	3440	4200	4990	3450
286	20900	23400	20500	22900	26700	20400	26800	32800	33200	21500	25400	24300	23900	22700	26200	22400	22600	19500	16700	23800	28000	24600	33100	35700	23100	22600	23900	28000	23600	21100	20400	29800	20000	21000	20500	29100	34400	32700	37100

Supplementary Table S1. Overview on the abundance of all relatively quantified 770 proteins

			Group->	HC	HC	HC	HC	HC	HC	HC	HC	HC	HC	HC	HC	HC	HC	HC	B1	B1	B1	B1	B1	B1	B1	B1	B1	B1	B1	B1	B1	B1	B1	B2B3	B2B3	B2B3	B2B3	B2B3	B2B3	B2B3	B2B3	B2B3	B2B3	B2B3	B2B3	B2B3	B2B3	B2B3
No.	Protein name	UniProtID	Gene	P01	P04	P07	P10	P13	P16	P19	P22	P25	P28	P31	P34	P37	P40	P43	P02	P05	P08	P11	P14	P17	P20	P23	P26	P29	P32	P35	P38	P41	P44	P03	P06	P09	P12	P15	P18	P21	P24	P27	P30	P33	P36	P39	P42	P45
535	Platelet glycoprotein V	P40197	GP5	16800	21600	20400	17700	16300	21000	15100	10800	14700	10800	13700	11700	13100	13400	22100	12100	18500	23600	20200	20900	20900	15300	13200	9440	12300	16700	15700	19800	17300	17300	22300	17700	16300	20100	18600	21300	12800	22400	15600	15100	15500	16000	23900	24800	23300
536	Pleckstrin homology domain-containing family D member 1	A6NEE1	PLEKHD1	199000	158000	218000	189000	156000	171000	137000	102000	129000	99000	142000	89800	118000	102000	233000	151000	183000	237000	194000	186000	190000	159000	121000	107000	178000	158000	189000	243000	143000	279000	244000	257000	176000	202000	162000	186000	112000	171000	211000	195000	185000	170000	224000	274000	323000
537	Plectin	Q15149	PLEC	67100	73900	68600	65600	72700	74200	66900	73300	63900	75800	68300	93100	96100	126000	115000	64000	64700	66600	65700	59900	70300	67600	68700	73300	72300	75100	79200	112000	116000	122000	71400	60200	80900	76400	66800	64300	64200	83100	75700	67400	81800	97200	103000	130000	113000
538	Plexin-A1	Q9UIW2	PLXNA1	1390	2020	2930	2820	2710	2060	4540	278	1680	277	6000	2730	1740	1330	4080	1920	1530	2080	7620	7070	3770	2990	8550	4580	3290	4180	4590	241	2370	7850	3430	4820	11800	7340	5440	5560	5570	1920	297	12100	21900	3480	4560	12000	14800
539	Plexin-A2	O75051	PLXNA2	28800	5900	14800	16400	24600	27000	19800	37200	3410	15900	4250	39500	35200	95200	14000	47800	9660	7190	3800	3930	13300	7760	6620	27800	19600	13100	8470	4360	64800	13400	6140	2780	2530	2230	4760	6630	1780	15700	4030	6630	3040	4820	4190	12000	6820
540	Plexin-A3	P51805	PLXNA3	16700	13100	7360	9500	9380	7820	3460	4770	1710	2750	2230	2820	3060	4580	3390	12600	12300	9600	4360	7010	9390	5740	2930	2320	1320	2140	1800	1840	2500	2720	8500	5010	8160	4160	5720	4630	5310	6650	1260	1650	949	4220	2000	2140	3060
541	Plexin-B2	O15031	PLXNB2	1900	2080	1960	1730	1550	1640	1990	1800	406	2290	2120	2090	3200	2070	3430	2200	2450	1590	2120	2080	2400	2520	3620	2580	684	1300	2270	1680	1970	2310	1570	1870	2100	989	1970	1560	6370	2370	1510	2120	1510	3520	4230	2930	1210
542	Poly(rC)-binding protein 1	Q15365	PCBP1	6020	7200	6490	6290	6490	6020	6150	6970	8960	6620	7630	8540	10100	11400	10200	5430	5440	7480	7360	7120	7420	7670	7710	7020	8540	9970	10600	9770	11600	11500	8010	6070	8050	6320	6870	8510	7000	9670	6910	9780	9670	8480	10900	12200	13400
543	Polycystic kidney disease protein 1-like 3	Q7Z443	PKD1L3	68300	87400	87300	67400	47500	73300	66900	71300	87700	67200	53400	49200	78700	67200	48300	78300	59700	124000	84000	81800	70600	52800	57300	61500	81000	83800	66400	90800	78100	99600	77700	73500	89200	74600	87500	132000	35100	92800	57400	82300	80100	60800	68100	82200	132000
544	Polypeptide N-acetylgalactosaminyltransferase 18	Q6P9A2	GALNT18	3520	4310	1750	2140	2930	1270	2040	2660	1520	2660	3370	2380	3430	1860	5050	2280	2810	447	462	1670	3060	2320	4070	1780	1020	1730	3490	3380	2090	3410	2520	1860	1620	920	1830	2460	3150	3390	2680	1770	2080	4710	4540	4000	2820
545	Potassium channel subfamily K member 13	Q9HB14	KCNK13	743	311	671	1100	478	110	921	828	1300	1060	575	1010	550	280	1240	1570	1480	784	599	865	344	915	952	1220	2570	739	1290	305	360	704	798	640	164	269	279	758	937	514	896	711	691	265	26	427	378
546	POTE ankyrin domain family member E	Q6S8J3	POTEE	1550	1700	1870	1570	948	1090	2080	2200	2510	2730	2230	2050	2560	2550	2090	1700	1910	1670	1360	1230	1710	2190	1580	2460	1670	2600	2260	1390	2350	2170	1350	1190	668	1280	1340	1780	1150	1740	1750	2170	1490	2220	1750	1420	1790
547	POTE ankyrin domain family member I	P0CG38	POTEI	314000	262000	337000	306000	337000	386000	239000	223000	290000	216000	265000	254000	357000	433000	517000	221000	294000	320000	342000	308000	320000	255000	302000	215000	266000	320000	362000	579000	499000	470000	382000	335000	339000	338000	312000	290000	237000	388000	332000	279000	325000	376000	530000	714000	478000
548	Pregnancy zone protein	P20742	PZP	101000	134000	159000	100000	136000	143000	107000	98800	494000	109000	149000	94900	124000	138000	146000	137000	121000	217000	211000	168000	108000	111000	202000	137000	114000	298000	129000	168000	129000	160000	204000	137000	150000	176000	140000	119000	118000	150000	203000	152000	168000	162000	135000	159000	547000
549	Pregnancy-specific beta-1-glycoprotein 1	P11464	PSG1	112	171	165	182	165	67	169	181	8060	93	206	215	157	77	142	121	162	173	165	230	187	100	304	263	496	268	210	136	213	94	108	235	218	249	205	210	289	148	212	241	208	60	141	102	206
550	Pregnancy-specific beta-1-glycoprotein 4	Q00888	PSG4	22	66	53	100	63	116	391	777	6380	2330	2860	3990	3120	1920	2100	15	50	84	52	201	184	1570	286	3460	354	2780	1210	762	1020	1490	21	21	23	8	167	166	1590	163	769	4330	495	2450	2680	228	1240
551	Pregnancy-specific beta-1-glycoprotein 9	Q00887	PSG9	1210	951	680	1150	1060	1150	1230	1420	2950	1580	1460	1660	992	1610	1770	754	785	596	658	1340	1200	1380	1780	1220	674	849	1130	2040	1330	1620	862	1150	3420	822	1080	1170	2530	1170	1020	1150	1120	1480	1800	1850	922
552	pre-mRNA 3' end processing protein WDR33	Q9C0J8	WDR33	102000	14000	101000	183000	98900	6120	304000	90800	60300	75600	156000	86000	138000	25700	125000	100000	109000	46600	185000	52100	43300	136000	361000	91500	13400	18300	178000	261000	42800	120000	136000	347000	180000	32500	18900	66100	842000	18300	123000	56400	151000	281000	333000	300000	32500
553	Probable E3 ubiquitin-protein ligase HECTD4	Q9Y4D8	HECTD4	8580	13000	6390	6800	10100	6700	6380	8580	4410	7350	9530	7880	9340	6190	10900	7180	7870	4100	5060	7200	8690	7130	13100	6450	4270	6000	7810	9930	6500	10600	8530	8050	10500	8090	7550	7950	11700	8380	7770	6350	7880	11100	11000	11400	9040
554	Probable guanine nucleotide exchange factor MCF2L2	Q86YR7	MCF2L2	6600	7080	8800	7060	6320	8450	6150	4860	7500	6770	6060	6240	6970	7830	9610	5240	7080	8400	7440	7690	8730	6430	5250	6730	6250	8580	7610	8550	8460	8260	8480	5950	5850	7270	7800	7630	3690	7510	7290	6030	7400	7410	9510	9880	9410
555	Probable non-functional immunoglobulin heavy variable 3-16	A0A0C4DH30	IGHV3-16	105	35	102	78	90	120	291	374	126	151	105	459	489	1680	216	330	62	97	54	81	80	137	35	312	224	220	117	56	1600	164	56	45	65	37	55	74	49	122	57	125	63	78	56	86	200
556	Probable non-functional T cell receptor beta variable 17	A0A087X0K7	TRBV17	2070	2260	1860	2120	1990	1990	1920	1920	2310	1970	2110	2030	2440	2390	2250	1500	1460	1800	2100	1880	2150	2150	2220	1970	1760	2680	1980	1730	1940	2130	1960	2050	2010	1830	2390	2240	2280	2340	1230	1630	1450	2490	2290	2540	2860
557	Probable non-functional T cell receptor beta variable 7-1	A0A0A6YYK4	TRBV7-1	5060	4140	4350	5010	5980	5920	5170	7730	3860	4240	3230	9660	9020	23100	6080	8630	2860	3830	3650	4130	4430	3720	4460	6950	4500	4500	4630	6450	14800	5440	4450	2140	4210	3300	3330	3510	3140	4860	3340	3140	4350	3930	5700	9180	4660
558	Probable non-functional T cell receptor beta variable 7-3	A0A075B6L6	TRBV7-3	25400	47500	42000	28300	41300	31700	51200	33500	14700	37300	40700	29900	44200	26400	47000	19000	22100	45000	59900	44200	31800	42100	45000	24600	43800	20700	50500	70300	49100	64700	35500	43500	34100	67900	47900	52300	63300	30000	75400	61500	64600	48200	51300	82800	60600
559	Probable serine carboxypeptidase CPVL	Q9H3G5	CPVL	88	97	112	83	37	97	138	175	201	95	101	192	148	267	186	139	69	135	82	54	119	77	53	178	216	206	104	235	277	235	89	52	934	148	101	143	9	98	68	111	171	19	161	72	415
560	Probable ubiquitin carboxyl-terminal hydrolase FAF-X	Q93008	USP9X	2020	2060	1760	2520	2870	1490	1960	2300	1760	2010	2450	964	909	470	2540	1060	1690	1490	2760	1520	1580	1920	5600	1380	1150	1180	2970	4190	565	2020	1720	3310	22400	2750	1550	1650	6000	1950	3510	1460	3370	4030	4180	5070	1360

561	Probable ubiquitin carboxyl-terminal hydrolase FAF-Y	O00507	USP9Y	2880	1590	1510	4180	2080	3290	2520	1870	4100	1000	1640	2450	2560	2560	6370	4240	3310	3080	1650	6230	2720	3010	3020	1510	2410	1960	4900	2880	1620	2480	3920	3620	3170	1230	2500	2870	4430	3820	3790	3800	1620	1770	3480	4090	3550
562	Proenkephalin-A	P01210	PENK	3180	2050	3270	4910	2720	934	3550	4620	5650	5990	4690	6610	6000	5060	8900	5050	5010	2900	1600	3210	2360	4340	6960	6560	10500	4810	9390	4400	5410	6900	3580	4470	3990	1560	1340	3350	7080	3840	5320	5600	6270	4770	2920	6090	4350
563	Profilin-1	P07737	PFN1	190	2880	558	191	733	1540	440	697	64	229	439	589	214	124	705	170	1020	749	189	4050	640	3230	1430	115	617	216	649	201	1480	334	536	711	496	2000	2290	633	871	4030	340	117	1510	541	1320	3960	2140
564	Progesterone-induced-blocking factor 1	Q8WXW3	PIBF1	30700	62500	56500	30400	45400	79000	50200	35200	52200	47100	54800	54600	66400	86000	85900	37300	57400	35500	46700	49800	75800	77900	47300	59100	38100	74300	54700	38300	72300	70400	36800	36000	38800	38000	69400	33600	47900	60800	33400	19700	21800	60600	96400	76100	65800
565	Prolyl endopeptidase	P48147	PREP	326	281	283	330	302	285	263	294	105	166	375	124	339	286	395	215	262	349	406	310	411	472	358	105	357	265	358	418	324	403	295	312	1060	334	181	440	315	381	205	376	288	299	360	408	226
566	Properdin	P27918	CFP	11600	11900	9810	12900	13600	14600	9680	8940	7510	7800	8920	9060	11200	10800	12600	8210	11200	11100	10200	9730	10200	10400	10600	7190	7050	6400	11400	11700	11700	15700	10600	10300	26500	8930	9920	8150	13000	11800	10100	8210	9930	14200	15300	15600	12100
567	Prostaglandin-H2 D-isomerase	P41222	PTGDS	877	961	789	878	778	898	1160	1470	1210	1210	939	1350	1210	2760	1090	1340	838	744	779	926	1110	889	965	1540	1130	886	698	823	2010	912	795	1200	1290	841	917	771	1210	928	671	867	903	1130	1060	1050	600
568	Proteasome subunit beta type-1	P20618	PSMB1	2810	4820	4180	2970	3050	3530	3640	3130	6360	2720	3290	3490	4450	3990	5880	3180	3750	4120	4200	3630	4120	3600	3390	3360	4120	4530	3960	6410	4450	7260	4220	3070	5390	5140	3620	4720	2170	3050	3120	3080	6180	3740	5960	6320	13400
569	Protein AMBP	P02760	AMBP	287000	391000	343000	254000	312000	361000	280000	261000	304000	271000	261000	277000	333000	421000	411000	279000	285000	290000	265000	308000	318000	271000	319000	247000	230000	314000	346000	290000	343000	378000	288000	286000	324000	281000	315000	278000	341000	384000	253000	326000	225000	398000	399000	469000	425000
570	Protein BUD31 homolog	P41223	BUD31	527	395	84	178	222	338	153	175	155	317	443	206	237	289	174	120	154	148	183	141	159	157	243	225	193	224	161	118	197	163	88	208	76	246	249	127	409	240	268	11300	179	297	207	180	222
571	Protein C3orf33	Q6P1S2	C3orf33	286	482	489	316	290	459	368	350	246	213	266	396	444	458	332	315	385	361	393	390	394	333	387	244	213	418	358	223	307	422	291	481	488	407	375	308	414	444	164	277	229	360	249	241	449
572	Protein CIP2A	Q8TCG1	CIP2A	24300	37000	24900	23600	27900	32500	18200	16200	10800	18600	16100	19200	24400	46900	37900	15400	22500	26000	29900	21600	25400	13300	20200	16500	25700	21500	22600	34700	49400	41300	29800	17000	15700	30500	25500	22800	10600	30600	21800	8590	23100	21000	29600	52000	38500
573	Protein Daple	Q9P219	CCDC88C	1230	1270	1410	1430	1310	1240	1250	1160	1310	1440	1310	1200	1160	1380	1520	1000	1190	1190	1260	1210	1350	1180	1240	1210	1520	1200	1420	1430	1550	1270	1060	1190	917	1170	1240	1320	1070	1450	1320	1170	1240	1110	1570	1550	1030
574	Protein FAM110D	Q8TAY7	FAM110D	6720	10300	15400	11600	4720	1700	24300	17500	22400	18100	11400	10600	19000	14100	11800	18600	11800	18600	5690	6980	7930	11400	14700	16100	33300	14000	21800	22100	18300	26000	14800	19600	16300	11700	5620	29500	10200	12700	24100	20700	31300	15300	12100	19600	29300
575	Protein FAM13A	O94988	FAM13A	13600	14200	13000	13000	12700	12500	12000	11700	13300	10900	14200	10600	13900	12800	34900	11300	11400	13700	14600	12900	12800	10900	14500	10200	12600	15500	13900	16700	12000	15600	13700	15100	18100	14700	13500	15100	13000	16200	11600	12500	13100	13000	14200	18900	18400
576	Protein FAM204A	Q9H8W3	FAM204A	1340	2890	1990	1080	1340	3060	1670	1740	1460	1470	1620	1220	1570	2720	1760	2370	1800	1810	1400	1830	1940	1710	750	1430	1590	2830	1590	1420	1870	1990	2000	2340	1540	1890	2260	1610	580	2530	1250	2610	1190	1890	1540	1650	4950
577	Protein FAM43B	Q6ZT52	FAM43B	12800	17100	3920	12400	7020	13500	10100	10500	8470	13300	10800	12800	4320	15200	26700	4420	6150	6430	2670	14400	8860	11700	9590	10100	5810	6630	7090	20500	13800	26000	4580	8520	6300	4260	9470	7700	16700	9700	4780	9460	5460	11900	14300	8200	9320
578	Protein FAM47A	Q5JRC9	FAM47A	19200	25100	15400	14500	18600	21800	17000	17700	24300	14700	19900	22600	26900	36000	32400	16900	17900	14500	13100	16300	15700	19300	12000	18600	8500	20200	18000	27000	33600	35800	15400	8560	9810	12000	17200	14200	17300	18100	13800	16300	12000	33600	38900	28600	41800
579	Protein FAM9A	Q8IZU1	FAM9A	466	344	415	539	442	194	886	504	526	494	23300	268	455	204	224	606	316	33800	399	37000	418	246	310	535	32400	38100	334	567	367	372	386	533	23700	510	954	34000	13100	490	767	1370	951	35600	252	141	342
580	Protein Hook homolog 1	Q9UJC3	HOOK1	9170	10200	8960	9070	8480	9420	9800	12900	16200	13500	14900	13000	16500	15700	17400	9320	11400	9270	6990	9620	10300	13600	9630	12900	6990	17900	13300	8930	15600	15600	8010	6300	5010	7130	11200	9030	8460	13700	9410	15700	9010	13900	13000	11200	17300
581	Protein KTI12 homolog	Q96EK9	KTI12	23400	26700	21400	26400	27000	27100	28800	28000	27900	26200	30700	25400	37200	30300	38000	20400	24500	22700	21400	18800	24600	26300	31700	25700	18000	29000	32000	31600	33300	39500	18600	25200	24200	19600	26500	26000	32300	29900	22200	28900	24500	30700	33000	36600	31600
582	Protein LBH	Q53QV2	LBH	180	46	112	220	16	201	154	114	367	94	255	171	218	168	301	62	18	77	171	55	140	164	410	136	161	205	246	283	97	15	25	127	98	16	140	197	280	192	194	278	174	189	346	26	111
583	Protein LTO1 homolog	Q8WV07	LTO1	543	817	1040	725	1190	909	1390	2110	1590	1940	2760	2050	2830	3660	4250	630	597	1150	1260	1470	1380	1740	3220	2330	4890	3390	3770	8500	6870	6090	1510	1620	2010	1330	1080	1720	2040	2590	4660	2760	5070	3740	4500	7060	6170
584	Protein Njmu-R1	Q9HAS0	C17orf75	3640	5180	4200	4890	2960	3060	6240	6500	11600	8680	5860	6880	6270	7570	6840	5050	4760	2960	3980	2470	3420	7700	6200	7040	4370	6660	5420	6000	5420	7570	4290	4070	2810	5140	3590	4720	9600	3380	6930	4190	5580	9250	7250	4180	7250
585	Protein O-mannosyl-transferase TMTC3	Q6ZXV5	TMTC3	1320	1440	1010	852	523	346	2140	2660	1570	4050	2050	2880	2790	2850	1650	2520	1610	515	1540	177	749	1770	542	3260	854	1510	976	1490	1710	2900	1630	1180	562	1480	439	989	1030	542	2180	953	3440	4120	1120	318	3000

586	Protein PBMUCL2	E2RYF7	HCG22	18	1	5	4	12		12	20	6	2	3	18	9	26		22	6	0	8	1	2	4	49	16	6	2	2	3	18	3	5	1040	1740	1	2	7	812		3	2	18		1	19	
587	Protein phosphatase 1 regulatory subunit 12C	Q9BZL4	PPP1R12C	3160	2220	2150	2750	2160	1570	2520	1930	3150	2210	3030	2510	3260	2420	2380	2200	1680	2390	2590	2020	2300	2910	2880	2200	2390	2910	3260	3700	2690	2180	2560	3080	3180	1490	2240	2980	3960	1970	1980	2750	2570	3060	3120	3430	2810
588	Protein piccolo	Q9Y6V0	PCLO	4000	3130	3320	3310	1680	829	5190	2870	2470	2820	3440	3930	3360	3560	3130	4230	3320	2690	4550	1910	2550	3470	4530	2500	2970	1670	5240	7060	3920	3820	4620	2920	13200	2710	1410	2080	4910	1560	3280	3710	6620	5550	5650	6800	2910
589	Protein prune homolog 2	Q8WUY3	PRUNE2	1550	2520	921	1570	2290	1100	2330	2270	692	1610	941	1610	1770	1490	1420	1640	1030	741	2810	671	636	1440	1500	1640	171	468	297	2270	782	1970	1250	11200	8250	4260	1110	811	1530	392	572	507	1600	2580	1830	740	909
590	Protein S100-A10	P60903	S100A10	460	212	1530	592	444	38	1130	753	1730	1180	537	802	961	719	1070	897	1070	983	1050	268	800	1020	388	1020	1500	700	1080	968	1040	1080	958	926	133	506	91	1110	347	315	1710	548	1580	966	1140	976	1250
591	Protein S100-A9	P06702	S100A9	307	234	592	338	199	122	646	236	869	318	1010	805	654	283	335	267	586	629	276	1520	290	1060	2300	419	858	218	2710	921	1580	342	333	485	1100	325	678	697	912	619	848	1260	910	392	401	1170	287
592	Protein S100-Z	Q8WXG8	S100Z	1190	333	685	836	1020	661	2070	2880	204	1100	438	3430	2410	8040	1160	3100	313	362	254	583	983	1300	585	2130	1800	700	906	1070	5740	1030	787	725	473	263	399	843	920	1450	321	1550	413	575	537	798	579
593	Protein shortage in chiasmata 1 ortholog	Q5VXU9	SHOC1	58800	17200	39700	49300	41600	42200	72500	148000	8920	67900	20700	176000	170000	395000	49500	157000	29600	22800	14500	12900	35100	31100	38100	120000	82200	38800	36100	31600	287000	51300	31200	15400	16200	10900	11300	23700	18700	41000	32300	28000	17600	17000	17900	64400	34500
594	Protein SOX-15	O60248	SOX15	5070	8700	3570	4830	5380	5770	4630	6400	3020	5570	5080	5480	5280	7250	4780	4520	5380	3570	6410	3590	4150	4800	4040	4580	2470	4690	3090	4550	4300	6360	5120	4480	4230	7330	5480	3730	3960	4410	4000	3460	4450	7010	5060	3320	5850
595	Protein TANC2	Q9HCD6	TANC2	2340	1830	1550	3020	2970	2340	1980	2390	1740	2330	3090	2520	2920	2450	5290	2390	2540	2440	2030	2770	1680	2200	2230	2370	3300	3050	3950	2800	2680	4390	2140	1940	1570	1410	2100	2270	2000	3560	1710	2540	1970	1770	1220	2850	2910
596	Protein timeless homolog	Q9UNS1	TIMELESS	1240	957	2920	2260	1670	1620	1370	1600	991	1350	1810	1810	2560	2530	5110	2620	2380	1490	1370	2000	1420	1840	1210	1600	2710	2090	2940	2560	4380	4130	1210	1020	576	925	1210	1440	1540	2170	1190	1640	1130	842	680	3220	2440
597	Protein TOPAZ1	Q8N9V7	TOPAZ1	6680	7860	6030	6630	6530	6510	6610	6070	7140	6350	7120	6340	5490	4680	6220	6380	6100	7060	6060	5810	6800	5640	5850	5950	4440	7560	5580	5860	4990	6290	6910	5320	7040	7390	6590	6030	5850	7150	5330	6110	5290	5290	5700	6490	9080
598	Protein tyrosine phosphatase domain-containing protein 1	A2A3K4	PTPDC1	550	547	753	546	513	792	653	885	565	830	421	1140	1000	2280	407	816	377	914	483	737	666	442	299	1030	792	921	413	515	1720	527	628	416	862	783	851	779	259	782	598	1840	454	321	235	304	767
599	Protein XRP2	O75695	RP2	5200	3400	5080	8630	19800		6400	6250	3340	5450	9310	5320	5770	13500	5100	5610	16200	62	4930	323	9320	11300	7030	6510	16000	8570	21400	11300	17500	4660	8590	3200	10900	4150	170	5610	19100	9880	12200	1160	9990	6480	12700	4840	1160
600	Protein Z-dependent protease inhibitor	Q9UK55	SERPINA10	112000	144000	114000	116000	124000	122000	116000	117000	115000	114000	112000	115000	128000	152000	135000	110000	121000	120000	118000	107000	111000	116000	110000	106000	96200	115000	111000	124000	131000	142000	119000	106000	95400	130000	119000	107000	115000	100000	101000	110000	111000	144000	140000	146000	157000
601	Protein-arginine deiminase type-2	Q9Y2J8	PADI2	38200	55800	43700	46000	53900	55300	45800	44600	55800	45400	54600	35800	40300	50200	48200	40200	46200	47300	48300	54400	55300	59600	57300	41000	38000	47800	35700	43400	46800	52700	47600	44700	51500	62000	51400	49300	74400	59700	51700	56100	41900	59800	44600	68200	76000
602	Proteoglycan 4	Q92954	PRG4	10200	8970	3450	9740	4920	8830	6570	7650	5750	5810	6740	6830	5120	14800	12800	4320	6060	5400	2130	9600	7550	11300	7070	5620	8010	6630	14000	12400	13000	10800	6000	7720	6340	5800	8080	7960	12800	8040	4920	9710	4280	6100	8690	6360	4300
603	Prothrombin	P00734	F2	569000	523000	498000	449000	457000	518000	368000	386000	366000	343000	424000	413000	526000	565000	650000	409000	523000	507000	467000	392000	474000	412000	399000	373000	396000	428000	564000	551000	513000	643000	427000	413000	384000	438000	411000	472000	361000	551000	385000	466000	313000	501000	586000	718000	644000
604	Protocadherin beta-15	Q9Y5E8	PCDHB15	5330	5290	5620	5280	5570	5160	6910	5490	7050	6160	4700	2970	5970	5490	6230	5580	3970	10200	6470	7090	5260	4110	4850	4650	9000	7570	7420	12200	6960	10200	6230	11000	11600	7190	7260	11300	2610	8780	6900	7820	9130	6050	5150	12000	10100
605	Protocadherin beta-3	Q9Y5E6	PCDHB3	3510	3730	3970	4340	5250	3820	5180	4030	2810	3650	4280	4060	6410	5280	7710	4180	3280	4730	5200	5100	4070	4420	5140	4100	6270	3890	7080	7050	6940	8370	4610	5610	5880	5860	4130	7790	5690	4540	8150	6970	6620	4750	5650	8930	6420
606	Protocadherin beta-5	Q9Y5E4	PCDHB5	6090	7160	6460	5980	6370	7750	7410	7650	8500	6880	9240	8660	8430	8510	8280	4940	6630	5740	5220	6180	6750	9750	5490	8030	3420	7550	6050	7150	7050	8110	5360	5140	3760	3870	7240	5530	7980	7930	5370	8050	4580	8480	9180	6530	9540
607	Protocadherin beta-6	Q9Y5E3	PCDHB6	29100	32400	27800	33200	44600	39600	28300	30200	28100	29200	33800	30900	36300	42500	52300	29900	32800	29400	27700	37800	31100	34800	34800	27900	40900	37100	38900	44500	45200	47100	33600	26900	46000	32400	32500	26600	36300	42500	28500	28000	35000	37400	36900	60200	52200
608	Protocadherin beta-9	Q9Y5E1	PCDHB9	677	842	351	968	641	504	767	682	481	948	678	576	358	908	1520	356	740	537	402	748	806	777	730	717	599	949	725	853	903	1500	648	600	210	370	432	712	897	632	358	765	638	619	923	761	845
609	Protocadherin Fat 3	Q8TDW7	FAT3	50000	112000	35900	38700	55000	79500	40200	73600	35200	66600	50400	65600	69900	99000	63700	67400	55600	36600	40300	26700	42100	41400	26400	55300	21400	56100	27600	39700	64000	83500	51000	33400	27100	70700	54900	32500	19400	55300	30200	33700	43200	82300	53000	29900	84600
610	PTB domain-containing engulfment adapter protein 1	Q9UBP9	GULP1	46100	52400	67000	49000	61700	53600	72200	43400	34000	47400	49800	37200	71600	52500	77000	45800	41500	67400	87400	70300	60300	58000	61600	45800	66900	37000	66500	93800	84100	101000	66000	91000	83400	103000	68300	83900	83000	41300	106000	89100	83500	68700	76600	112000	102000
611	Putative beta-actin-like protein 3	Q9BYX7	POTEKP	3530	3750	2740	2600	1940	1700	4000	5270	2420	5070	4420	5040	4990	5670	2880	5790	4170	2700	3240	1560	2260	3430	3760	4630	1790	3090	2250	2810	3830	4480	3270	3070	1890	3190	1960	2460	4190	3100	3310	3150	5840	6170	4130	1300	3660
612	Putative deoxyribonuclease TATDN3	Q17R31	TATDN3	5140	4670	5890	4120	3400	4710	4040	3370	5260	4740	3890	4840	5260	6950	6890	5210	5610	6520	3990	5060	5890	5080	2670	4180	4880	6030	5180	4660	7340	5570	5690	3830	2410	3670	4560	4840	3330	4850	4530	6140	4090	4470	5380	5520	7900

613	Putative glycine N-acyltransferase-like protein 1B	A0A0U1RQE8	GLYATL1B	6080	7010	7580	6110	4780	7310	6450	5940	6450	6460	5880	7860	5850	9010	9210	4160	4940	19500	13200	11100	7750	15600	6250	6040	128000	6720	12700	103000	9970	14100	7560	24600	32600	21900	6840	22000	13600	6400	24300	25900	38500	6370	11900	10800	24000
614	Putative histone H2B type 2-C	Q6DN03	H2BC20P	319	75	46	46	144	13	167	186	41	827	175	168	219	131	58	548	93	18	128	13	36	242	107	360	6	136	26	17	15	91	185	77		165	91	95	93	5	95	31	268	395	92		74
615	Putative inactive neutral ceramidase B	P0C7U1	ASAH2B	15900	13200	15300	13900	18400	13400	10700	11100	5580	8410	13700	8610	12900	10400	15100	11000	15100	17600	20900	14300	12900	13700	20700	9310	12500	6640	18300	35400	13700	32300	16800	19300	35000	28200	11900	16100	33000	8880	39300	15900	25800	19400	21400	39100	17900
616	Putative inactive phosphatidylinositol 4-kinase alpha-like protein P1	Q8N8J0	PI4KAP1	20200	9800	10100	14700	14400	17800	22000	16500	37800	18100	29100	24300	36000	55300	41400	7010	32500	14700	14200	28900	10700	29000	9440	33400	64400	8210	23000	27400	47100	31400	16400	11500	7340	8320	17200	18300	11700	38000	22200	21300	19700	18900	11600	34000	36200
617	Putative protein ATP11AUN	Q6ZP68	ATP11AUN	19900	38000	14400	20100	23200	23800	18700	25400	13300	27200	19900	21200	23900	31300	22700	23100	21900	14800	20500	12500	16700	17700	15300	21400	7280	19100	10700	19700	17400	29700	20800	20900	17000	31000	23100	14600	15000	15000	14700	14400	21600	32500	24200	14400	26800
618	Putative protein FAM10A4	Q8IZP2	ST13P4	11	5	4	7	7		11	6	8	6	8	1	36	12	12	5	8	37	16	29	15	6	9	8	5	11	8	183	47	12	19	65	234	4	7	47	77	43	14	13	24	7	91		14
619	Putative protein FAM47C	Q5HY64	FAM47C	33		63	42	34		288	448	1870	1400	492	284	1120	299	610	83	10	168	25	22	36	247	240	957	1880	1360	1160	1470	758	1050	25	33	1	43	41	437	63	376	1180	1730	1480	359	488	471	928
620	Putative protein FAM47D	A6NHR8	FAM47DP	1270	4300	1390	1520	1340	3120	2910	4710	2410	5440	2870	2640	3600	10300	4340	2750	2370	1750	1700	1350	2550	3180	941	3250	1320	4760	1830	2270	4430	5800	2040	1400	464	3570	2970	2120	405	3230	2010	2560	4030	4400	1310	1110	8840
621	Putative protein ZNF321	Q8N8H1	ZNF321P	743		1750	309	191	22	246			8	16	579			7	3		10000	5260	1220	391	6340	610	4	54800	4	3430	67600	52	2060	1290	19100	25400	12300	58	9960	4080		5910	9810	20700	6	2470	1410	7470
622	Putative uncharacterized protein	Q86SY8	KTN1-AS1	3800	4140	3280	1550	3510	2430	4730	1940	2050	1950	2210	2730	2940	1340	2970	3320	2660	515	527	4250	3160	3900	761	1320	2530	3110	2960	2120	4410	4300	3990	2060	2280	917	3620	2210	1090	2600	1190	25000	1610	3380	3060	3110	4430
623	Putative uncharacterized protein KTN1-AS1	Q8N3U1	LOC400692	1790	2720	1340	1760	2130	2140	1920	2650	1910	2780	2500	2230	2540	2490	2320	1930	1690	1450	2030	1470	1630	2020	1850	2520	795	2000	1450	2230	2210	3490	1130	1670	2480	2530	2280	1470	1170	1430	1550	1680	1120	3680	2510	1630	2920
624	Putative zinc finger protein 75C	Q92670	ZNF75CP	6580	8420	7940	7180	9090	8880	7520	8620	8200	7630	9350	8240	9350	10100	10100	7280	7020	8700	8320	8760	9060	7680	8600	8080	10600	9960	8750	11700	12400	11300	8900	7990	8920	9660	9440	8690	8570	9400	10000	9940	9980	8970	8390	11200	12500
625	Pyrin domain-containing protein 2	Q56P42	PYDC2	1810	4030	2740	1440	2000	4720	1830	2070	1810	443	1650	1690	1300	1570	1090	2260	2000	2540	2420	1380	2530	519	424	1350	3290	2890	978	2200	1650	1800	2710	738	4590	3830	3290	1840	347	2720	1180	1460	3470	412	1330	1080	9550
626	Rab GTPase-binding effector protein 1	Q15276	RABEP1	3030	2320	3070	2920	2470	2240	3120	3200	2930	3030	3580	3150	3040	2450	3500	2900	3150	2870	2920	2650	2950	3290	2800	2730	2260	3200	3000	2910	2740	3060	2920	2900	9300	2650	2620	2720	4080	2750	2730	2390	2810	3210	3500	3020	3100
627	Rab15 effector protein	Q6BDI9	REP15	4290	5440	10400	5220	1660	5430	8590	6180	11500	8740	6100	8380	11200	16700	18200	6650	7280	9780	7070	5990	9550	8590	2620	7540	14800	11700	11100	13600	19600	15100	9740	5580	2110	6920	4960	9830	1450	14100	9100	11200	10300	10500	14800	11800	24300
628	RasGAP-activating-like protein 1	O95294	RASAL1	638	1100	1010	495	854	993	177	32	142	46	90	31	57	68	117	307	717	768	632	637	508	76	239	49	153	95	199	185	45	79	1040	966	1160	417	535	454	258	358	198	119	118	59	168	433	72
629	Ras-related GTP-binding protein A	Q7L523	RRAGA	6630	7070	6990	7750	7480	8190	6810	9360	5380	5890	5570	8950	10900	15900	11300	6480	5860	7830	6410	8480	8430	6880	8280	7360	8440	6330	8550	15200	14200	11700	8360	6610	5420	6600	7180	9000	6890	8980	4380	6720	7910	8340	12200	15300	9340
630	Ras-related GTP-binding protein B	Q5VZM2	RRAGB	9350	8400	9230	6600	9140	11700	7570	9770	6480	7700	10400	8950	12200	10200	12400	9200	9930	13400	9320	10400	9400	10300	5390	9510	12700	9100	12100	15900	14000	24300	9350	8670	8510	16500	9590	10300	5440	9590	22900	14000	15000	11200	11300	14300	21800
631	RB1-inducible coiled-coil protein 1	Q8TDY2	RB1CC1	4320	4460	3370	3380	4500	3940	3440	3640	2970	3330	4190	4070	2950	5960	5070	3060	3680	3410	2440	4070	4000	3430	3600	3310	2900	3720	2740	5330	4950	5320	3480	3980	3990	2770	3090	3090	4200	3310	2810	3010	3110	3780	3290	3880	4140
632	Receptor-type tyrosine-protein phosphatase F	P10586	PTPRF	16100	12100	15000	14500	13100	12600	12000	9610	7310	7670	8620	4570	5220	6570	6640	15800	11700	25800	14400	14800	12000	6260	9480	5770	9880	10400	7680	8990	6790	10400	16400	17900	24800	12300	14200	20600	4420	15900	8770	8730	10800	7090	5120	14400	11400
633	Receptor-type tyrosine-protein phosphatase S	Q13332	PTPRS	181	562	226	188	115	138	98	133	293	178	89	283	186	401	28	170	112	220	171	160	183	106	79	247	279	367	167	69	219	84	201	156	1260	154	210	199	96	210	83	215	76	107	154	36	245
634	Ret finger protein-like 2	O75678	RFPL2	1430	626	2000	958	1310	1150	901	894	690	283	499	422	835	719	1070	981	941	2940	2690	1640	1650	1520	1330	449	1320	850	2050	1670	1990	1710	1760	1330	677	1730	1300	2390	1560	1190	1730	1120	1810	844	1040	1640	820
635	Retinol-binding protein 4	P02753	RBP4	93800	74800	54400	78300	68300	83800	50500	46300	31300	38400	43500	49600	65800	68600	80500	73000	81100	81200	47600	63700	86000	73300	55700	28300	34100	51300	74600	35700	61100	63300	64500	54200	75000	43000	57400	64600	70900	81000	21200	51900	22100	63500	52400	71400	62100
636	Rho family-interacting cell polarization regulator 1	Q6ZS17	RIPOR1	118	24	33	56	47	17	48	33	87	71	43	54	16	27		90	82	33	15	42	20	62	82	40	71	23	27	23	27	4	215	74	988	13	24	18	34	7	25	161	31	296	55	46	
637	Rho GTPase-activating protein 20	Q9P2F6	ARHGAP20	1660	3080	2520	2060	1990	3770	1900	2080	1700	1560	1490	1850	1990	2760	2180	1890	2700	1970	2140	1860	3090	1900	1630	1980	1990	1730	1640	1330	2510	2480	1810	1240	1780	2090	2510	1730	936	3870	1590	2120	679	1910	1970	2950	2030

638	Rho GTPase-activating protein 9	Q9BRR9	ARHGAP9	1240	653	786	503	625	458	418	287	257	223	281	170	288	250	244	940	883	789	507	897	831	459	491	332	182	162	334	720	269	317	1180	830	629	454	434	561	900	273	341	113	101	613	226	501	207
639	Rho guanine nucleotide exchange factor 5	Q12774	ARHGEF5	62900	85600	66000	45000	62200	70600	39900	48200	56400	31700	46300	29300	43100	50600	59500	52600	62000	60800	54300	66100	59700	46500	49100	35000	52200	65300	50300	54800	70600	65900	57100	49900	51800	65800	57400	52500	37400	76700	52800	42700	43600	39700	40700	77800	115000
640	Rho guanine nucleotide exchange factor 9	O43307	ARHGEF9	819	1460	1420	1050	1620	930	1090	897	1260	797	1200	1320	1440	1340	1820	929	934	1780	2000	1740	1450	1390	1820	1210	1880	1190	1830	4390	3080	4760	2110	2010	2570	2270	1100	1820	806	1100	1930	1400	2930	2130	2360	4660	4400
641	Rhodopsin kinase GRK7	Q8WTQ7	GRK7	7350	6290	7970	8570	7320	4910	9570	9210	8280	7650	10100	10100	9630	7380	10500	7790	9090	6590	6840	8150	7720	11300	8570	7940	4710	6290	9930	9740	8480	10400	8300	6900	4340	4360	6410	7420	12400	7020	6250	8980	6160	12600	12500	11100	7090
642	RNA exonuclease 1 homolog	Q8N1G1	REXO1	10000	10300	12800	12400	9200	15800	9800	7830	8570	11300	8770	10300	12800	14400	19300	5500	9980	10300	17900	10500	13400	9050	5830	8390	5700	11800	10500	15500	17200	17000	11700	7260	4690	14700	9930	12700	4710	12300	11400	7770	9440	13900	23200	25800	9790
643	RNA-binding motif protein_ X chromosome	P38159	RBMX	5700	10500	7680	7020	11300	11800	7360	7630	5100	7180	7090	8140	9580	19100	8900	7690	7180	7870	7770	8230	9330	8220	7160	7260	7190	8700	7060	5480	15900	9390	7470	6820	6200	8770	9120	6330	6030	10400	4020	6780	4220	9070	7930	9290	14200
644	Rootletin	Q5TZA2	CROCC	38000	54600	35600	37300	43500	43600	34600	33900	35100	36100	37500	33400	43200	47900	55400	36000	37700	32600	40900	30700	37900	37100	37600	31800	26000	34900	36600	55800	45300	61200	40600	36900	59700	46900	42000	36500	37400	38000	36600	36700	42200	58600	62300	51200	57000
645	Rotatin	Q86VV8	RTTN	57000	67100	73800	63700	60100	73200	47400	34600	40300	37800	39700	38000	31100	48400	56200	41600	66800	78600	68200	69400	72400	44400	36500	35300	45000	52900	40000	53800	57300	54700	78400	55500	50800	64600	65300	62800	22400	64600	44300	37400	41700	45300	52600	79100	64300
646	Roundabout homolog 1	Q9Y6N7	ROBO1	2430	1100	2830	2190	1940	1210	2030	983	1980	1150	1410	1410	1210	1340	1730	1830	1220	3510	1380	2840	1720	2160	785	2040	2280	1300	2410	3820	2710	1740	3260	1050	651	1250	1750	1900	587	674	1560	2190	2130	1600	3510	5390	1250
647	S-adenosylmethionine decarboxylase proenzyme	P17707	AMD1	3560	5540	3380	4850	6110	4220	3660	3520	5330	3800	4030	4640	4270	4690	8510	5500	5530	3800	3270	5150	2890	3830	3860	3760	6340	5610	4890	3790	4500	5980	3480	3930	3440	3430	4150	3300	3420	4310	2900	3550	3870	3100	2480	5530	5530
648	S-adenosylmethionine synthase isoform type-2	P31153	MAT2A	1670	1620	930	1780	1210	2120	1430	1420	2420	1840	1400	1730	866	3030	3430	679	947	1200	623	2150	1530	1900	1290	1680	1310	1210	941	2660	2680	3570	845	1290	678	1140	1540	1450	2730	1500	886	1620	1020	1610	2130	578	1480
649	Scm-like with four MBT domains protein 1	Q9UHJ3	SFMBT1	2900	2990	1840	3010	2420	3480	3330	3750	3410	3180	3280	4070	2560	6550	6150	1870	1830	1930	1870	4040	3500	3880	3120	3390	3480	2750	2730	6400	5340	5830	2530	2890	3380	2600	3310	3220	4820	3600	2560	3500	3180	3230	4290	4000	5180
650	Scm-like with four MBT domains protein 2	Q5VUG0	SFMBT2	347	103	378	205	350	147	170	79	21	71	214	29	24		30	137	249	440	629	315	206	232	621	113	103	5	255	874	47	441	453	727	1490	1000	206	365	1510	13	1420	244	801	262	392	751	189
651	Secreted Ly-6/uPAR-related protein 1	P55000	SLURP1	10300	18900	7260	9560	9940	12800	8940	13100	5650	10300	9210	9690	8650	13800	7770	12100	11000	7320	11900	6290	8090	8840	7080	8030	3700	8760	5040	8310	8310	12300	10900	7780	6050	15200	11000	7010	6470	8360	6890	5920	8340	13000	8550	6730	11900
652	Selenoprotein P	P49908	SELENOP	22100	19800	29600	26900	19200	12800	30900	28000	24100	27900	28600	24200	32100	32800	42200	30900	30800	30500	32700	23700	26000	29800	34100	25500	35000	25700	36500	39800	38400	44800	30400	32500	31300	27800	17000	33600	45600	28100	29900	26100	30800	36800	38700	56200	48100
653	Semaphorin-4B	Q9NPR2	SEMA4B	34700	37700	34800	32800	28200	36000	22200	23500	30400	22500	22600	24600	26900	27700	38600	27300	31600	34900	34200	31900	35900	30300	31000	27200	30100	27000	36700	39100	33400	38700	28500	34100	39800	35600	30900	38000	38200	35800	32100	37000	31100	31900	37500	50400	46200
654	Serglycin	P10124	SRGN	1010	1720	1530	661	1360	1860	655	433	627	190	102	126	259	334	410	927	1270	1410	830	1080	1210	321	338	190	419	255	275	398	221	82	942	734	310	978	1040	794	61	1040	350	106	283	86	539	172	90
655	Serine/arginine-rich splicing factor 1	Q07955	SRSF1	2830	3420	5660	2770	1390	1700	5810	5660	5430	6290	3820	5700	7580	8850	8420	4140	4970	4000	3470	2950	3750	4390	6030	5590	4930	4980	8240	2400	7500	7240	3100	4580	2480	2630	2250	4410	4490	4900	4250	5560	3820	7450	6930	5730	6160
656	Serine/threonine-protein kinase	O95747	OXSR1	1410	611	2130	1300	1370	1150	1270	926	1440	424	1240	857	1750	1010	1910	1020	1070	3010	3070	2020	1900	1750	1900	980	1710	1520	2860	2550	3100	2740	1810	1670	1120	2140	1550	2850	2040	1670	2360	1970	2650	821	2100	2740	1790
657	Serine/threonine-protein kinase SMG1	Q96Q15	SMG1	42900	47000	47600	50300	50700	55100	48600	43700	48100	50800	53300	53400	67900	68500	86300	32500	45800	51000	53000	47100	55900	57800	48600	47800	43800	56400	64100	79900	73000	90900	42800	42200	51000	45600	50200	54000	45100	59200	53500	49600	51500	72100	84200	95600	82700
658	Serine/threonine-protein kinase tousled-like 2	Q86UE8	TLK2	354	1080	891	770	515	282	1160	1490	1100	1550	1090	934	977	395	481	1020	871	586	1150	315	670	834	1300	997	488	630	469	852	234	1060	688	967	679	1360	634	818	1060	143	870	249	2100	2020	1100	560	1630
659	Serine/threonine-protein kinase ULK4	Q96C45	ULK4	9000	9330	5390	3120	4990	4460	3690	2900	2650	2700	7140	3060	4050	3170	3480	5880	4890	4910	6210	3880	3620	3710	4040	3280	3500	3610	3260	5620	3140	4000	5840	5630	10800	8710	4390	3070	11900	3720	4260	98600	6150	5260	3880	4410	7510
660	Serine/threonine-protein kinase WNK1	Q9H4A3	WNK1	3030	3260	2660	3290	3340	2300	2240	2800	2520	2940	3670	3980	4480	3670	7560	2440	2660	2630	2460	2380	2270	2800	4270	2640	5360	3660	5310	5950	4590	6510	2180	2390	3060	2730	2300	2400	2980	3850	2940	2750	5610	4380	4260	7170	4700
661	Serotransferrin	P02787	TF	69900	65900	48700	63600	69900	72800	61000	87000	891000	91200	52800	86900	93400	153000	73400	89300	43500	45500	87100	48300	60300	81300	56200	160000	91700	65300	52700	51000	124000	56100	54400	66500	65700	40200	183000	48600	73400	84900	47200	91700	44400	58900	90800	63400	52100
662	Serum amyloid A-1 protein	P0DJI8	SAA1	15700	22200	20900	18000	20600	21400	13400	17000	21100	26300	24800	24400	16500	26000	33400	12800	14400	27200	17400	30300	20700	18100	15500	20400	35400	20300	43000	474000	37700	37800	26200	135000	112000	94900	20100	31800	58100	21800	301000	147000	197000	28200	43200	37600	80200

663	Serum amyloid A-2 protein	P0DJI9	SAA2	3340	3250	3450	3000	3330	3600	2640	2850	3040	3850	3200	5230	2320	3890	2180	3690	2660	5340	2770	7490	2910	2780	2460	2840	5960	3480	2410	59800	3020	2460	5540	25000	16400	4370	3410	3710	3880	3650	41000	6040	69900	3530	4760	2860	14200
664	Serum amyloid A-4 protein	P35542	SAA4	41400	36500	31900	36400	31900	36600	35200	38700	54300	40600	40600	48200	38000	43600	54900	37400	33000	51100	26400	43300	35600	45900	41400	40900	47600	42800	66000	89400	56500	57000	41500	60900	33400	37700	31500	36800	71700	50700	66900	61900	34100	46000	58900	81100	80900
665	Serum amyloid P-component	P02743	APCS	67500	60100	70200	53600	85600	73000	53000	43500	46700	36500	49500	48900	74600	76100	103000	47100	46400	85800	84200	68000	73600	71300	58800	42300	58600	63300	97600	119000	112000	128000	77000	67900	91000	84200	62400	78700	71900	83500	73000	71200	74100	70800	94500	135000	98200
666	Serum paraoxonase/arylesterase 1	P27169	PON1	99400	225000	260000	242000	191000	229000	170000	205000	165000	240000	173000	279000	361000	407000	337000	166000	216000	198000	163000	184000	158000	273000	148000	230000	159000	233000	272000	127000	169000	220000	178000	184000	64600	91200	151000	195000	183000	202000	97100	122000	104000	321000	322000	247000	222000
667	Serum paraoxonase/lactonase 3	Q15166	PON3	43300	70300	38900	40500	45100	45900	37400	45800	33700	40600	40400	42000	38900	49700	42000	46400	48100	34500	43600	28300	37500	41300	28200	37200	33400	31600	31000	55400	33900	46300	43500	37600	28500	53800	41000	32000	38100	32000	34600	31300	36500	57700	47900	35700	44900
668	Sex hormone-binding globulin	P04278	SHBG	29300	45500	34200	23000	29400	43100	44500	46500	115000	48700	51500	34100	38300	35900	38800	53400	41200	20800	36700	26700	24600	45900	32500	56200	22800	135000	24600	34800	25900	42600	31400	22000	34600	35300	34800	16400	35400	26100	39300	33400	32200	75100	32000	40000	178000
669	SH2B adapter protein 1	Q9NRF2	SH2B1	19100	26300	18900	11400	8620	12700	14200	16600	12900	13700	12800	12200	10700	16700	14000	20000	19400	17300	9240	8240	15800	14200	8260	13400	7430	13900	6940	8910	12000	16800	18300	13700	8230	11600	10400	12600	14000	17400	8870	13800	7850	11100	13000	9980	25900
670	SH2B adapter protein 2	O14492	SH2B2	10	111	602	101	13	22	561	509	281	273	1190	48	206	28	763	7	152	203	816	699	27	1140	21	76	20	94	247	93	78	775	924		106	480	701	395	49			39	2040	12	146	213	187
671	Short transient receptor potential channel 4-associated protein	Q8TEL6	TRPC4AP	10700	12500	6750	9980	14300	13000	9470	13500	6730	12600	17400	14000	13900	19700	17400	6600	10900	9210	10000	8850	10500	11400	8390	10100	8270	11400	20200	8140	23300	18200	7400	4940	12100	10500	9150	9410	8720	18500	6120	9600	6210	14800	18900	15400	19000
672	Signal-induced proliferation-associated 1-like protein 1	O43166	SIPA1L1	42500	47100	42200	42500	47900	46400	40200	44300	50900	48700	59900	52200	79900	71600	90100	50300	40500	41500	45100	45600	45800	49000	52300	52900	57700	54100	62800	90300	73200	87400	44200	46300	56400	48600	42700	41500	65000	49800	55100	70400	73900	73000	83000	89700	80400
673	Slit homolog 2 protein	O94813	SLIT2	19500	37000	17200	17600	25900	25800	18100	21100	9780	17800	21400	12800	17100	14700	18100	23100	18600	16600	22700	17800	17500	19600	20300	11600	6990	19200	14000	20700	12500	24800	25500	17500	17400	27700	22900	14800	21900	21600	13000	12500	17700	33600	24400	20900	30100
674	SPARC	P09486	SPARC	4320	3920	2900	3370	3100	3330	3570	6290	7040	7140	8890	7040	9750	10300	15800	4200	4120	5920	3250	3230	3670	6090	6070	6470	7730	7540	11200	10100	13700	10000	3290	5540	5340	3260	3580	3450	4700	4450	6230	8360	8240	8080	11300	12500	12000
675	Sperm flagellar protein 2	Q9C093	SPEF2	6310	6310	7280	4050	4520	5390	4190	4090	2460	3230	1820	3730	3980	7690	3490	6690	6520	5340	4580	4010	4610	2550	2650	3600	2360	3140	2490	1620	4890	3560	4900	5580	4210	4340	4620	3230	2180	4100	1770	1560	1980	5000	3390	3810	3920
676	Sperm protein associated with the nucleus on the X chromosome D	Q9BXN6	SPANXD	849	417	467	2700	359	471	744	630	720	1070	501	970	383	2040	979	546	361	376	294	884	675	799	723	652	431	309	311	1470	1260	908	1190	637	109000	249	582	652	2660	283	379	1560	758	526	597	351	191
677	Sperm protein associated with the nucleus on the X chromosome N1	Q5VSR9	SPANXN1	501	452	556	287	197	212	406	128	342	310	641	68	256	128	498	273	306	719	645	604	530	500	398	141	439	593	672	746	499	656	792	523	324	797	546	705	97	492	1230	2030	671	269	417	659	1050
678	Spindle assembly abnormal protein 6 homolog	Q6UVJ0	SASS6	18900	25700	23200	23800	26200	33700	17300	15200	14200	15900	29800	18100	24200	15600	41000	14500	26200	22500	21800	22900	30500	30700	23000	11900	12400	21800	33900	35200	17700	51100	19100	17400	17000	20800	26800	19100	36700	29900	20800	20300	19600	46400	42200	44900	44400
679	Spliceosome RNA helicase DDX39B	Q13838	DDX39B	1960	2330	3380	3140	1280	1380	3370	3270	12800	4070	3330	2730	2370	4050	4100	2660	1890	3180	2280	327	2160	4120	2870	2520	1360	6350	3060	2690	2020	3380	2640	2870	2860	2600	1930	4080	5690	3660	6470	4180	5000	3940	2180	4140	8210
680	Squamous cell carcinoma antigen recognized by T-cells 3	Q15020	SART3	6320	10600	6940	7430	8150	9150	6950	6870	9100	6510	6900	6020	7580	9250	8940	6220	7110	6900	6770	7360	7730	7720	7350	6020	5270	8930	7420	9030	7820	9300	7260	7330	9020	8380	8400	7640	7300	8630	7530	7860	6740	7670	8910	8840	12700
681	SRR1-like protein	Q9UH36	SRRD	45700	9030	54500	52300	17800	2020	67100	58000	39000	47500	67700	77000	85200	102000	93700	70300	51200	38000	47400	8550	45400	57000	48900	50900	53300	47800	72700	105000	95200	76400	65400	30900	38800	24700	8690	29700	53400	52300	68900	68700	64300	114000	101000	91400	107000
682	SS18-like protein 2	Q9UHA2	SS18L2	4270	5700	1290	6400	4170	5160	4840	4730	2450	4000	5180	4400	1560	4360	6900	1120	1860	1690	1090	5700	3380	5430	9070	2490	612	1010	2560	7770	3580	7010	1610	5700	5730	1910	4180	3150	20800	1920	1890	2090	1750	6670	6790	3180	767
683	Sterile alpha motif domain-containing protein 11	Q96NU1	SAMD11	810	219	868	906	873	372	1050	1030	1180	971	717	574	924	893	667	1090	485	1590	777	961	679	857	807	815	1580	512	1170	2500	992	1190	1060	859	1870	1020	749	2350	517	478	1280	1080	1600	768	747	1820	395
684	Striated muscle preferentially expressed protein kinase	Q15772	SPEG	8850	6100	4830	9220	10000	5380	4970	4640	7210	6950	4950	5560	14600	6370	11700	7570	8130	4850	6250	13300	9490	9510	8660	4610	28700	9280	6150	29100	5140	5580	11000	11000	10700	19900	9310	11600	11900	10600	9560	10700	12200	13600	10500	6370	13100
685	Structural maintenance of chromosomes protein 1B	Q8NDV3	SMC1B	48900	108000	38500	34500	43400	79500	28500	53300	18300	33300	24900	30600	35200	55200	35700	85900	59300	41600	37500	24300	40500	26000	18500	27900	18700	33600	17800	23200	29600	48200	54200	38900	24000	69000	51800	29300	16900	59200	18700	20100	34800	40000	19700	25100	74100
686	Succinate dehydrogenase [ubiquinone] iron-sulfur subunit_ mitochondrial	P21912	SDHB	2300	1530	2080	1420	1300	1190	579	526	686	451	1280	644	342	441	551	1720	1420	1590	1480	1020	958	541	2140	391	553	1610	547	678	493	431	2730	1380	2050	1510	1180	749	625	823	1960	1530	762	735	811	1120	541
687	Sulfhydryl oxidase 1	O00391	QSOX1	3160	3760	3230	4280	3730	3050	2920	4740	3680	4890	4710	5960	6380	6980	8860	3450	3480	3570	2150	3060	3290	3660	3110	4420	6700	5750	5820	4220	7020	6990	2450	2350	1640	2330	2750	3010	3910	6620	3630	5020	4450	5170	3650	4220	5010

688	Surfeit locus protein 2	Q15527	SURF2	501	146	505	477	232	140	653	264	417	482	159	399	322	452	252	561	173	615	242	268	234	439	91	645	405	406	362	380	687	200	772	136	39	150	223	239	69	12	302	104	443	357	469	435	41
689	Symplekin	Q92797	SYMPK	1460	2010	1530	1150	865	1100	1150	725	1460	1250	975	1020	1620	1550	2030	1120	1340	1930	1340	1360	1660	2510	473	1070	1810	1560	1910	2030	1810	1560	1310	1150	391	1200	1090	1490	625	1460	1190	1350	1300	1260	1790	1680	1980
690	Synaptonemal complex protein 3	Q8IZU3	SYCP3	802	194	218	683	464	463	340	258	231	255	510	216	509	67	626	344	511	336	283	366	726	695	883	159	106	202	850	227	188	312	185	350	1430	80	297	416	860	514	99	373	45	430	420	517	123
691	Syntaxin-12	Q86Y82	STX12	14	88	32	65	47	101	58	3	7	14	12	15			8	4	90	118	61	96	135	13	16		109	48	20				92	49	23	119	86	57		36	130	61	39				
692	Syntaxin-19	Q8N4C7	STX19	26800	45100	36400	29700	39400	42900	28300	26000	33800	24800	33900	27400	38600	41700	44800	23900	31800	32900	34400	36300	34800	25400	33700	28300	25100	34000	32700	19700	35100	39800	35900	33100	36000	33400	38000	27400	21900	42100	32800	31300	26700	38000	36300	35900	49400
693	T cell receptor alpha variable 14/delta variable 4	A0A0A6YYC5	TRAV14DV4	1210	755	1430	1860	1350	212	3050	2180	1120	2990	1980	1180	1780	424	974	2230	1580	1300	2910	653	896	1330	3230	1820	1040	1270	1340	1670	456	1930	1890	5050	2920	1480	573	1740	4130	1050	2060	1290	4050	4220	2380	1280	1190
694	T cell receptor alpha variable 8-3	A0A0A6YYJ7	TRAV8-3	532	1250	1640	890	265	2520	1510	1720	3880	2760	2310	3290	3260	7820	4300	1140	1480	766	874	996	3070	3350	677	3060	2490	6290	1950	703	4990	2870	633	469	164	582	1740	852	229	5330	794	868	544	1930	3680	1840	4720
695	T cell receptor beta variable 24-1	A0A075B6N3	TRBV24-1	5410	11000	7170	6920	5580	732	12900	23800	7160	16000	12900	13400	16700	14200	9320	9710	9890	5010	6840	1600	6830	14200	9640	10300	2100	5380	7650	7320	6640	17100	6510	7040	4100	10700	2390	6120	15100	4430	7840	4850	13900	29700	16900	5110	13000
696	T-complex protein 1 subunit zeta-2	Q92526	CCT6B	5560	6920	5200	6740	6570	6740	5350	5600	6700	7020	6140	7150	8150	15100	17000	6410	7020	6270	5090	5590	5040	6210	4910	6240	9300	8910	7290	9570	14600	16200	4590	4860	3160	4840	5010	4720	4120	7510	5520	6020	5930	6950	7410	11900	20100
697	Testican-1	Q08629	SPOCK1	2510	3420	2860	2570	2840	3010	3160	3290	4590	3450	3770	4310	5350	5930	6010	2430	2070	3070	3080	2950	3350	3410	3120	3860	4170	4720	4760	4990	5610	5600	2660	2810	4180	3100	3300	3720	3250	4260	3090	4580	3570	3930	4840	5320	5860
698	Testis-expressed protein 12	Q9BXU0	TEX12	2710	2600	2930	2990	1740	1260	1810	1990	1440	2040	1450	1370	1280	2940	877	3860	2510	4500	2860	2450	1550	1320	1960	1840	2230	1700	1540	1810	2300	1040	3400	3630	3020	2620	2110	3400	1430	1890	2250	2630	1880	1090	921	1900	1180
699	Testis-expressed protein 2	Q8IWB9	TEX2	19300	18900	31800	26500	15900	14100	28900	23100	34500	26500	22300	21200	27900	28100	38300	19900	25200	32700	27500	25700	22800	26600	32100	22100	27300	22300	31800	34000	34800	38400	24000	32800	21900	23600	18800	33200	35000	20400	30800	21700	32500	32200	40300	45700	36300
700	Testis-expressed protein 29	Q8N6K0	TEX29	1440	2370	1550	1160	2270	1010	1990	461	1220	1410	1930	1150	1090	396	996	1190	1950	212	95	2800	2230	1880	3140	1010	1290	2200	1250	783	1950	1410	1770	1570	2280	535	1950	1310	2780	1790	1260	1680	759	1900	1450	1920	2630
701	Tetranectin	P05452	CLEC3B	42300	43400	29700	36100	44800	42100	33300	41100	18400	31500	39600	31300	41800	52800	56400	29700	32200	23500	23300	27800	34900	32300	40600	26000	19300	24800	28900	36600	46900	39700	24000	24600	30800	27400	28100	23500	37200	40100	18500	18500	22600	43500	44400	45900	23800
702	Tetratricopeptide repeat protein 21B	Q7Z4L5	TTC21B	29700	35100	28400	25300	24200	25600	27500	25900	23300	24400	26300	25200	28800	34500	31200	28000	27900	28400	25900	24500	27600	27200	22300	23600	29200	26600	28900	26000	38300	35800	28400	23800	23300	28100	25800	26200	30700	27100	26000	30700	26000	28400	29700	31600	39900
703	Tetratricopeptide repeat protein 33	Q6PID6	TTC33	24600	33700	32900	25400	25000	20100	30900	29000	19100	23400	26700	25200	32000	35400	36100	33700	29600	31300	39200	26000	27100	29500	37500	23700	27900	23900	33400	40700	39200	44000	36200	38500	42900	40800	21200	29100	44800	34000	29700	24500	34200	37500	36800	60900	65200
704	Tetratricopeptide repeat protein 9A	Q92623	TTC9	20300	2510	10500	11800	24800	28900	22100	57400	3690	24000	5830	71400	61100	238000	16800	37200	7480	7530	4490	3950	11700	7890	4650	45800	38100	19300	8940	5100	151000	16200	4820	2310	3250	2430	3490	6480	2430	19400	4790	10300	2560	4330	4780	10800	9020
705	Tetratricopeptide repeat protein 9B	Q8N6N2	TTC9B	13500	19000	31800	24000	12100	9380	33700	26000	41800	31600	26200	30000	34100	33500	42100	18400	26200	26500	30000	20800	24100	27700	35200	31900	42200	25600	40800	44300	43400	45200	25600	23100	13100	20900	14500	33500	26900	25600	47500	32400	45000	39200	47400	50600	45200
706	Thioredoxin domain-containing protein 9	O14530	TXNDC9	2730	2530	2220	2380	2220	2340	2280	2100	3090	1950	2620	2040	1760	813	1770	1760	3010	1870	2110	2580	2280	2450	2010	1850	510	1510	2140	1390	1150	1380	2390	1300	1120	1500	2560	1940	2820	1840	1670	2080	1550	2290	2980	1740	833
707	THO complex subunit 5 homolog	Q13769	THOC5	378	518	834	500	64	40	1160	1060	1720	742	362	812	538	312	492	894	574	1060	345	410	94	510	1260	823	1890	401	3960	1530	1070	857	1040	2680	472	588	76	436	3330	130	3180	2200	1050	811	799	218	2230
708	Thrombospondin type-1 domain-containing protein 4	Q6ZMP0	THSD4	2500	3300	1450	1670	2710	2270	2150	4980	1100	3220	1690	6550	5060	16100	2890	5380	1370	1130	1090	964	1710	1610	2750	5030	3500	1650	2710	2890	13700	2260	3750	1360	1820	621	838	1070	1250	2620	2780	2140	802	2360	2640	6840	2510
709	Thrombospondin-1	P07996	THBS1	72700	101000	88200	79200	88600	76200	89100	70800	82000	76300	79600	76600	91300	94100	115000	71400	90500	100000	92400	87800	94600	77500	88500	67000	71900	70800	90600	108000	106000	104000	97300	84300	98100	104000	81600	93500	78000	104000	90000	68900	96800	106000	150000	117000	135000
710	Thyroglobulin	P01266	TG	4780	5330	6650	6330	4820	7730	2350	1570	1400	1090	1920	1960	2170	2850	1330	4370	6530	3580	4270	3870	6490	3000	2860	1620	1410	2100	1780	1320	2210	1510	4730	3000	29200	2060	4680	1670	2320	2860	1410	1070	1220	1480	1950	1540	1800
711	Thyroxine-binding globulin	P05543	SERPINA7	52400	84900	61400	67600	71600	70600	85700	75000	120000	77200	92500	80500	102000	127000	112000	68600	72600	39800	68900	63800	67000	80200	115000	86100	63200	99100	99900	101000	118000	121000	66300	72000	97500	80700	46300	58900	131000	84600	79300	85800	80200	132000	106000	160000	151000
712	Titin	Q8WZ42	TTN	376000	466000	339000	365000	420000	458000	413000	486000	391000	444000	443000	486000	528000	688000	558000	400000	374000	340000	360000	331000	393000	430000	373000	430000	356000	433000	399000	504000	573000	635000	329000	343000	334000	393000	395000	368000	418000	436000	362000	408000	386000	579000	526000	514000	634000

713	TM2 domain-containing protein 1	Q9BX74	TM2D1	1800	16	2720	851	2470	859	17	363	304	2330	286	1780	69	1140	182	731	1240	1570	85	58	5650	2890	924	2730	4600	98	4150	1160	3810	4490	13	3540	642	1790	1200	3460	128	1970	6710	8310	53	4370	2010	1650	225
714	Transcription cofactor vestigial-like protein 2	Q8N8G2	VGLL2	1750	1500	1330	1910	2090	1210	993	1330	407	855	1190	694	743	790	821	1860	1760	1190	980	1310	1110	1350	3610	656	973	570	1780	1470	852	799	2320	695	2530	1690	999	733	6030	582	266	715	594	3010	1750	3970	398
715	Transcriptional repressor scratch 2	Q9NQ03	SCRT2	3440	5000	3600	3640	5060	5240	3280	4050	1830	3870	5340	4140	4280	4650	5000	2180	4000	4820	4290	4300	4410	4820	4550	3880	5700	4100	5140	5990	5860	10200	3790	4360	7830	7500	3780	4360	5920	4810	9180	6090	6000	4770	5450	6870	11000
716	Transforming growth factor beta regulator 1	Q3YBR2	TBRG1	979	121	699	885	136	106	1950	734	708	967	1590	899	2270	567	2250	1570	622	122	1220	105	177	686	246	1360	684	303	1680	2420	1030	900	898	2130	491	88	124	269	3460	196	1550	12400	1770	2080	2500	410	1010
717	Transforming growth factor-beta-induced protein ig-h3	Q15582	TGFBI	55300	46000	61200	56200	46600	45100	76100	86200	88300	75100	54500	81000	116000	126000	83100	66700	68400	48000	54600	34800	58300	65800	58500	85700	54500	74200	85100	56900	109000	75600	40900	56100	37100	37900	38700	60700	76200	59200	57400	71400	43500	65300	65300	67200	79600
718	Transgelin-2	P37802	TAGLN2	483	91	428	448	503	232	392	292	383	444	646	273	330	158	487	428	482	495	578	534	598	607	529	427	584	479	429	611	339	642	583	532	331	617	486	514	686	335	537	626	616	663	463	543	646
719	Transient receptor potential cation channel subfamily M member 6	Q9BX84	TRPM6	102000	128000	107000	85100	106000	115000	104000	124000	144000	111000	107000	154000	142000	205000	144000	123000	108000	98800	74700	89900	95500	116000	82300	123000	73400	121000	110000	91900	171000	143000	97000	74000	72300	67500	95500	82700	113000	107000	86100	120000	70700	140000	142000	127000	194000
720	Translationally-controlled tumor protein	P13693	TPT1	3930	1920	4620	4190	1670	2410	4340	3440	4800	3450	3920	4090	4390	4270	8740	5510	3710	6130	7870	4800	5330	4990	4930	3420	5500	2980	6190	11900	5310	9800	5910	8710	3860	11200	3540	10700	5010	5320	13300	5350	21000	3800	10600	8640	6550
721	Transmembrane and death domain protein 1	P0DPE3	TMDD1	4250	4920	3980	3470	4910	4060	3850	3290	1100	3320	3870	3790	5500	3410	5130	3060	4570	2810	4450	3120	3510	4370	5370	4020	2300	1670	3990	4700	3570	6150	3780	4010	7440	3010	3610	3030	9800	3290	2490	2040	3380	7540	9460	7480	3040
722	Transthyretin	P02766	TTR	469000	322000	281000	367000	338000	338000	290000	277000	183000	248000	302000	299000	393000	369000	497000	347000	367000	339000	211000	268000	423000	445000	285000	210000	200000	278000	402000	233000	382000	390000	268000	212000	326000	170000	261000	283000	457000	432000	143000	311000	152000	428000	406000	428000	402000
723	Trinucleotide repeat-containing gene 18 protein	O15417	TNRC18	549	598	702	1100	780	747	1320	927	888	891	884	790	1100	1440	1090	801	698	642	1060	728	847	1310	1250	996	671	951	924	690	1220	898	452	1020	9060	637	837	556	2240	737	836	339	742	1030	1560	888	796
724	Trinucleotide repeat-containing gene 6A protein	Q8NDV7	TNRC6A	33900	35500	27600	36900	50800	40900	30700	35500	28900	30600	33800	37500	42300	49500	76200	41300	39300	34400	28600	37700	27500	34800	32100	31400	45100	33700	47700	45400	48400	65000	28300	29900	34800	28800	32000	28700	36700	43700	26000	31300	31600	35300	23800	56400	51600
725	tRNA (cytosine(38)-C(5))-methyltransferase	O14717	TRDMT1	23500	23200	21900	20700	20500	28400	20200	19700	24500	14100	20600	24900	42200	27600	36500	13300	22900	21100	20700	20800	26800	23600	29200	18100	15800	24400	28200	27500	23400	37200	23500	22800	34800	23700	20200	23500	32200	26900	10300	21000	17000	24600	31500	46000	43900
726	Tropomyosin alpha-3 chain	P06753	TPM3	6540	6590	5140	7460	8700	7820	6330	7040	7850	7610	8720	9260	7660	7790	11700	7170	8510	6100	5310	7730	5200	6840	6840	7470	7690	9590	7940	6300	6370	8580	5380	5400	4820	4860	6970	4810	7280	8270	5010	6650	5620	6420	4880	8240	7960
727	Tropomyosin alpha-4 chain	P67936	TPM4	6620	12700	10700	17400	6130	28800	7560	8800	19700	5230	4330	7940	6920	9140	10900	15800	17400	18100	4800	28300	13400	6690	5810	5540	4670	14800	10300	3240	6670	4490	19300	15700	3930	2980	15600	9680	8500	29000	5410	15100	2310	5230	7440	5680	10900
728	Tyrosine-protein kinase Blk	P51451	BLK	16700	18600	14600	11700	12200	15400	13000	12800	15700	9170	13200	11700	9440	11200	10500	13200	14700	11300	9580	11500	12900	16000	8860	9340	5210	12300	8100	7190	9070	9620	14100	10900	8000	7250	12700	10500	16000	13700	7340	11900	5350	11000	11000	9130	10300
729	Tyrosine-protein phosphatase non-receptor type 20	Q4JDL3	PTPN20	3860	5800	5950	3910	4160	5600	4830	5400	5630	3510	4950	5410	6020	6310	6490	3990	4400	5690	5580	5460	5930	4690	4680	4470	5800	7120	5120	9790	7190	9230	5680	4620	5910	8170	5360	6540	2920	6100	4500	5020	9700	5380	7480	9320	18900
730	U5 small nuclear ribonucleoprotein 200 kDa helicase	O75643	SNRNP200	2590	3040	3870	3880	3230	4400	3420	3770	3860	3900	4840	3720	4180	4100	4410	2720	4080	3460	3770	4010	3620	4190	3940	3560	4500	3790	3870	5380	4130	5120	3610	3770	5000	4310	3860	3160	5750	4330	6130	5840	5990	4760	4690	5390	6250
731	Ubiquitin conjugation factor E4 B	O95155	UBE4B	113000	183000	93900	102000	132000	121000	101000	106000	95200	125000	121000	124000	169000	192000	199000	123000	114000	88100	115000	76500	98500	98700	87600	110000	80600	118000	117000	184000	151000	234000	113000	97600	105000	157000	117000	89300	108000	95400	87200	106000	156000	211000	202000	159000	222000
732	Ubiquitin D	O15205	UBD	3170	315	239	1220	1030	85	1180	1470	705	1110	1310	1460	1620	2480	1880	4290	1240	474	859	87	870	957	612	1200	201	1120	646	1970	1340	1920	1620	1550	194	1680	735	1200	782	187	456	507	1030	1950	1730	130	1620
733	Uncharacterized protein C16orf78	Q8WTQ4	C16orf78	18700	46300	29500	23400	31100	35300	22000	18900	19800	21600	22500	19400	20800	28400	30300	15500	26200	30800	31500	27500	25100	20400	22000	19000	21100	23900	22500	28600	28700	30400	27000	29900	30000	31600	30800	23800	20700	29700	30600	23900	23000	24500	25600	45400	36500
734	Uncharacterized protein C3orf20	Q8ND61	C3orf20	4120	1310	1980	4720	2450	3720	8040	8850	10600	12900	19400	10800	15000	4730	24700	2330	2910	2560	2220	4270	6660	19000	23400	9390	8560	11800	23400	9660	8270	18500	1270	1740	3620	1410	4200	6720	36300	11600	6880	14100	5840	25900	24700	23900	9460
735	Uncharacterized protein C9orf153	Q5TBE3	C9orf153	125		51	116	286	178	593	1740		534		2140	1310	4250	7	385	26	7			26	14	3	1560	701	130			2550	31								30							
736	Uncharacterized protein KIAA0825	Q8IV33	KIAA0825	38500	52800	41300	50800	49900	43100	45900	38900	46300	47400	49100	54400	59700	62500	76900	30800	42600	40600	48000	45600	46000	48600	59400	44200	53500	36700	55100	89900	68800	76100	49000	38800	65100	54100	40000	47800	79600	50800	52200	33400	66700	76900	96800	113000	66900
737	Unconventional myosin-Vb	Q9ULV0	MYO5B	3970	4420	6370	5440	5540	4310	5270	2190	4210	3220	3310	3240	4390	4790	9200	2830	4270	6660	6480	6540	4520	4400	3920	3540	5800	3560	5360	8110	6080	7940	5990	5640	5150	6290	3920	5580	3810	3600	3700	3180	5610	5160	8930	12900	7010
738	Unconventional myosin-XV	Q9UKN7	MYO15A	34000	42000	33700	31100	46700	37200	33400	37700	31600	36500	40600	38100	52300	73600	61400	32300	42400	21600	32200	31200	50700	40100	28600	43400	31400	50900	39400	34700	58900	55400	34100	32900	64500	37000	36100	31700	27700	52000	29700	24400	33900	38800	45200	34300	52000
739	Unconventional myosin-XVB	Q96JP2	MYO15B	382	227	214	256	128	42	339	408	218	329	388	480	233	192	553	508	528	393	186	455	284	363	297	344	749	423	391	178	254	270	406	515	75	150	283	265	109	404	236	185	174	151	44	205	281

740	Unconventional myosin-XVIIIa	Q92614	MYO18A	2740	2270	2170	2170	3190	3010	2000	1750	1440	1770	2590	1520	1980	1280	2880	1970	2240	2630	2010	2630	2800	2190	2080	1400	2380	2080	2870	3900	2300	3850	2870	2390	16900	2580	2160	2330	1750	3230	2430	2080	2750	3020	2360	4840	4210
741	UPF0545 protein C22orf39	Q6P5X5	C22orf39	3	2	4	40	3	2	4	1	3	3	1	6	7	5	5	2	4	4	5	3	4	2	2	3	0	6	5	1	4	16	1	5	10400	3	3	3	12	2	6	3	4	3	4	3	2
742	UPF0606 protein KIAA1549	Q9HCM3	KIAA1549	46200	68200	69400	52800	52900	52800	44600	33200	45000	36300	34100	33100	47400	54700	61000	42700	55800	62500	63700	48900	54600	40700	43900	34000	40400	39100	47400	64500	59800	61000	62300	71600	52300	61000	50800	55100	38600	48000	47800	36100	42300	50500	65000	75200	65000
743	Uracil-DNA glycosylase	P13051	UNG	5180	4620	6350	6070	6400	4890	6240	6340	6760	6640	9320	6650	8260	7340	11200	4490	5900	5970	7580	5360	6990	6250	7430	6060	8580	7330	8760	14700	8360	11400	5300	6710	7160	6540	6330	6910	7210	7000	8410	7500	7690	7800	10900	14500	12400
744	Uridine diphosphate glucose pyrophosphatase NUDT22	Q9BRQ3	NUDT22	2600	4180	4310	4390	3400	7990	5600	3910	7320	7150	7930	7810	9970	12900	13500	3490	4720	2970	3530	4860	8040	11700	5770	8480	5770	11000	8050	4350	10800	9620	2650	2620	1080	3690	6020	3590	5390	7570	4580	3360	2390	8980	17300	9860	7030
745	Uridine-cytidine kinase 2	Q9BZX2	UCK2	7660	13200	6860	9170	8040	13700	33000	65000	49000	88600	59900	78300	86700	120000	76300	13500	8900	7420	9030	9050	16300	51500	22300	69800	25400	71100	40000	54600	77400	95600	8500	5810	2250	14700	16100	18600	18000	40000	37300	52100	56300	86900	69800	32500	106000
746	Vacuolar protein sorting-associated protein 13A	Q96RL7	VPS13A	31400	27100	72000	48700	23900	19300	66000	52600	83900	59900	51900	57400	75100	84900	103000	40400	55500	58400	64900	45400	58800	56000	65000	61500	90200	64000	83400	91500	108000	94100	55400	68200	43600	41900	31300	68000	57200	57800	85800	61200	86100	76200	93600	117000	110000
747	Vasopressin V2 receptor	P30518	AVPR2	4350	4100	2950	3910	4470	5270	3370	3750	4730	4680	4780	5190	5340	10900	11600	5780	5280	4190	2930	4420	3200	4260	2640	4680	9030	6520	5890	5530	9050	10000	3540	2430	1780	3480	3810	3260	2300	6130	3620	5130	4350	3650	3030	6220	9980
748	Vitamin D-binding protein	P02774	GC	1280000	1510000	1250000	1200000	1200000	1210000	1420000	1450000	1810000	1380000	1450000	1260000	1860000	2000000	2250000	1130000	1300000	1040000	1180000	968000	1360000	1380000	1550000	1260000	1150000	1430000	1670000	1760000	2060000	2440000	1130000	1260000	1370000	1300000	852000	1280000	1710000	1510000	1520000	1240000	1650000	2220000	2040000	2630000	3120000
749	Vitamin K-dependent protein C	P04070	PROC	7210	8290	8240	6670	6660	6870	6950	5860	5080	4760	5450	5320	7290	8510	6200	6160	6530	8220	8210	8510	7760	5340	7950	4850	6710	6330	9500	6730	8940	7630	7840	9740	10400	7580	6930	7990	8990	9120	6670	9770	6580	6190	6580	11100	10300
750	Vitamin K-dependent protein S	P07225	PROS1	88300	96200	82600	96200	86400	97600	87600	87100	75500	88300	116000	87700	102000	109000	180000	72400	85600	98000	91300	111000	105000	122000	117000	82500	109000	75700	122000	182000	127000	189000	82900	118000	130000	113000	85500	110000	189000	114000	156000	114000	147000	137000	170000	178000	130000
751	Vitamin K-dependent protein Z	P22891	PROZ	8200	4590	7170	7650	8410	10700	8020	14200	7300	8420	4560	15800	13700	34300	6530	14000	5020	5560	5050	4130	7160	5260	4940	12300	9680	9540	6520	4390	24000	5220	4190	4890	4690	5860	4840	5820	4070	8370	5300	6540	4930	5040	5830	5170	6190
752	Vitronectin	P04004	VTN	955000	1090000	1060000	820000	666000	963000	868000	778000	1200000	807000	918000	863000	1080000	1170000	1300000	875000	978000	1060000	749000	805000	925000	860000	760000	806000	845000	1240000	1070000	1190000	1270000	1250000	1080000	839000	865000	898000	885000	962000	871000	1160000	1030000	1040000	907000	1210000	1280000	1550000	1950000
753	Voltage-dependent anion-selective channel protein 1	P21796	VDAC1	1020	512	633	307	352	234	250	286	66	238	329	509	332	1610	281	821	427	712	123	319	246	20	188	333	281	122	56	55	899	36	795	274	109	286	248	125	128	371	212	309	105	24	572	16	43
754	von Willebrand factor A domain-containing protein 8	A3KMH1	VWA8	21900	24900	21000	15500	27000	14400	27700	27900	21100	27500	31100	34200	42300	21700	45100	17900	21000	2420	2970	28200	29800	29900	31700	25700	21100	27100	34500	35600	42300	44500	21900	22100	29600	10000	23700	27200	29800	36200	23200	32200	24200	39400	42800	41200	44500
755	V-type proton ATPase subunit E 1	P36543	ATP6V1E1	20700	25700	21400	19300	24300	23700	21000	18600	18700	19700	18100	9900	20300	17300	24800	17100	15800	33500	24000	22200	19800	14200	24400	12500	26800	24900	26100	42400	19100	38600	23700	23700	34500	31300	27200	37400	11500	33700	26900	23900	33500	23500	21100	46200	37100
756	WASH complex subunit 4	Q2M389	WASHC4	4710	6830	5180	4760	3790	5490	5140	6340	7380	6260	7070	8100	8090	12200	7810	4620	4790	5220	4020	4850	5820	5470	3320	6350	5440	8530	5720	5320	10000	7930	4580	3580	2130	3400	5500	4940	5000	7840	4490	7640	4390	6810	8080	5710	11600
757	WD repeat-containing protein 31	Q8NA23	WDR31	60	87	609	151	221	108	340	128	20	130	503	158	25	8	100	190	93	376	1430	1140	370	360	1900	539	1070	219	240	3530	219	741	899	2080	5320	3520	657	392	1790	4	5910	1660	6470	391	292	2190	2480
758	Whirlin	Q9P202	WHRN	16600	8570	12900	23700	27300	10300	13000	6080	6660	8240	9610	5350	7800	4790	11200	6270	10700	8370	26200	12000	9600	9680	53700	5800	5690	4980	12400	27000	6710	9460	16300	79000	96700	18500	9630	9780	53300	5870	14400	5130	16000	16600	27100	46700	5490
759	Zinc finger and BTB domain-containing protein 47	Q9UFB7	ZBTB47	621	1430	475	483	539	226	460	598	250	449	453	489	504	635	515	757	583	420	675	361	467	561	350	323	161	408	396	348	303	428	715	417	324	769	563	387	400	562	215	236	133	671	494	286	475
760	Zinc finger BED domain-containing protein 6	P86452	ZBED6	3060	1620	3220	4180	2580	934	4530	3790	3490	2930	5060	3870	4370	1410	3730	2750	4010	2180	3460	2780	2570	4840	4610	3360	446	1740	3880	4460	1760	3530	3380	2840	1390	1270	1780	2770	7620	1120	2950	2420	2440	6390	6400	4680	1260
761	Zinc finger CCCH domain-containing protein 15	Q8WU90	ZC3H15	3670	13800	7430	6570	4290	1190	12100	25300	7420	19200	8850	14600	18300	26900	7900	13100	10300	4040	10600	1730	4950	16000	5750	13500	4630	9660	4020	16700	8990	22100	10100	11600	4130	16800	3920	10700	12700	3130	13300	7910	24900	42300	17800	3570	31700
762	Zinc finger homeobox protein 3	Q15911	ZFHX3	14200	36200	16600	9490	12500	14900	10900	10300	12100	8910	13000	11500	12400	13300	11700	13500	13800	12200	10500	10500	11100	11500	11800	10200	5970	13200	10800	9940	12400	12100	14000	18600	13500	9230	12100	9030	19600	15100	9380	11400	7400	13700	13400	12800	16500
763	Zinc finger protein 211	Q13398	ZNF211	110000	173000	130000	133000	166000	184000	137000	146000	209000	137000	185000	194000	212000	198000	219000	95300	137000	120000	92400	145000	139000	187000	126000	160000	64000	185000	150000	162000	175000	227000	119000	99200	77000	78800	150000	102000	192000	143000	108000	179000	94800	218000	232000	174000	254000
764	Zinc finger protein 469	Q96JG9	ZNF469	1240	516	1040	1090	1240	792	1080	1040	1390	1080	1190	1220	1130	899	1300	1030	913	771	739	1040	1020	1320	1860	1110	676	969	1120	1520	880	1050	1200	794	2510	662	922	835	1920	1140	1090	1260	1240	1470	2010	1860	868

765	Zinc finger protein 484	Q5JVG2	ZNF484	1280	597	1310	954	657	288	1430	1140	1660	1440	1510	1380	1360	675	1120	1600	1020	1760	1470	1090	750	1740	1270	1300	2490	1400	2400	4110	1010	3960	1590	1990	2990	2140	583	2030	4200	732	5600	2290	3430	1930	1560	1880	3050
766	Zinc finger protein 532	Q9HCE3	ZNF532	2500	892	4620	2220	5350	2920	1040	2180	1280	2730	2090	2560	1390	3610	1450	1700	3490	2910	840	1310	8020	8640	1730	3370	10800	1610	4330	3350	7790	10300	923	4910	1160	4800	2750	5120	861	4190	10200	8060	1340	10100	5800	5200	1580
767	Zinc finger protein 827	Q17R98	ZNF827	19900	15700	22500	25400	20800	11900	32400	23500	22500	20400	28300	21000	26000	16000	22300	19300	21700	17700	24900	15900	15500	27200	38200	22800	9220	12900	26200	29900	15300	23000	23400	22200	21200	15300	14900	19200	67600	12900	20800	15000	21800	40400	38900	35200	17000
768	Zinc finger SWIM domain-containing protein 5	Q9P217	ZSWIM5	2070	1820	2630	2300	2450	2140	1600	1060	1270	1170	1250	1220	1690	1970	2290	1460	1990	2480	2420	2310	2630	1720	1640	1150	1790	1380	1730	2840	2420	1990	2620	2640	2640	2460	1870	2090	1400	2090	1830	1670	1670	1680	2440	3010	1890
769	Zinc finger transcription factor Trps1	Q9UHF7	TRPS1	2130	2660	3370	2700	2550	2400	3060	2180	4760	3050	3130	2460	2830	2100	3450	2060	2750	2960	2760	2560	2290	3050	2620	3310	3280	2940	3660	3480	2500	3740	3060	2690	2500	3130	2350	2880	3040	1920	4570	2870	3620	3740	3830	4360	4050
770	Zinc-alpha-2-glycoprotein	P25311	AZGP1	160000	114000	105000	166000	204000	154000	130000	147000	98500	147000	224000	207000	198000	216000	238000	99300	107000	102000	127000	109000	155000	185000	211000	114000	109000	117000	223000	277000	209000	305000	110000	87400	248000	136000	115000	126000	527000	142000	221000	180000	178000	273000	268000	371000	185000
