# Supplementary material for: Unveiling Distinct Proteomic Signatures in Complicated Crohn’s Disease That Could Predict the Disease Course
Source: Int J Mol Sci. 2023 Nov 30;24(23):16966. doi: 10.3390/ijms242316966 (PMC10707401; doi:10.3390/ijms242316966)
Supplement: Supplementary file 1 [file ijms-24-16966-s001.zip › Supplementary methods_LC-MSE and search parameters.pdf]

Supplementary methods. LC-MS^E and search parameters

Description of experiment settings for LC-MS^E analysis and identification of proteins

**LC-Parameters**	
LC gradient<br>A: 0.1 % formic acid in water<br>B: 0.1 % formic acid in acetonitrile	0min-5% B-5m-5%-95m-40%-100m-85%-105m-85%-106m-5%-120m-5%
Flow rate	0.300µL/min
Column temperature	55°C
**MS^E-Parameters**	
Lock spray	GluFib precursor 785.8426 m/z
Acquisition times	10-110min
Acquisition mode	positive, resolution
Mass range	50-2000Da
Capillary voltage	3kV
Sampling cone	40V
Source offset	80V
Source temperature	80°C
Nebuliser gas flow	6.5 bar
Trap collision energy	4V
Transfer collision energy	2V

**Protein search parameter**	**Settings**
Name of peak list-generating software and release version (number or date)	Progenesis QI v3.0 (Waters Corporation)
Name of the search engine and release version (number or date)	MS^E built in search engine of Progenesis QI v2.0 (Waters Corporation)
Name of database searched and release version/date	Swissprot database release 2022/03 limited to human entries
Enzyme specificity considered	Fully tryptic
# of missed cleavages permitted	1
Fixed modification(s) (including residue specificity)	carbamidomethylation of cysteine
Variable modification(s) (including residue specificity)	oxidation of methionine
Fragment ions per protein	5
Fragment ions per peptide	2
Threshold score for accepting protein identification	≥1 significant peptides
Threshold score/E-value for accepting *individual* MS/MS Spectra	False discovery rate at peptide level <4%
software/method used to evaluate site assignment	No PTM reported

Presentation of Protein Identification Results

**Information requested**	**Reported**
Accession number	UniProtID number and Protein name
Number of *unique* (in terms of amino acid sequence) peptides identified	Only *non-conflicting* peptides have been considered (Progenesis QI v3.0)
