# Supplementary material for: Unveiling Distinct Proteomic Signatures in Complicated Crohn’s Disease That Could Predict the Disease Course
Source: Int J Mol Sci. 2023 Nov 30;24(23):16966. doi: 10.3390/ijms242316966 (PMC10707401; doi:10.3390/ijms242316966)
Supplement: Supplementary file 1 [file ijms-24-16966-s001.zip › Supplementary Table S2. Overview on the VIP scores PLS-DA, t-test and foldchanges of the 24 significantly different proteins among the CD and HC groups.pdf]

Supplementary Table S2. Overview on the VIP scores PLS-DA, t-test and foldchanges of the 24 significantly different proteins among the CD and HC groups

				VIPScore PLS-DA			t-Test				Fold change	
No.	Protein name	UniProtID	Gene	Comp. 1	Comp. 2	Comp. 3	t.stat	p.value	- log10(p)	FDR	CD vs HC	HC vs CD
1	Complement factor H-related protein 5	Q9BXR6	CFHR5	5.00	4.16	3.73	4.3421	8.52E-05	4.0697	0.006482	**9.95**	0.10
2	WD repeat-containing protein 31	Q8NA23	WDR31	6.84	5.74	5.08	4.7565	3.47E-05	4.4602	0.002931	**8.78**	0.11
3	Haptoglobin	P00738	HP	4.73	3.92	3.57	5.1244	8.80E-06	5.0553	0.002931	**7.84**	0.13
4	Serum amyloid A-1 protein	P0DJI8	SAA1	2.46	2.06	2.00	3.8795	0.000433	3.3631	0.020613	**3.36**	0.30
5	Putative glycine N-acyltransferase-like protein 1B	A0A0U1RQE8	GLYATL1B	2.61	2.17	2.06	4.7764	3.37E-05	4.4726	0.002931	**3.14**	0.32
6	C-reactive protein	P02741	CRP	2.49	2.06	1.91	4.1453	0.000175	3.7574	0.010234	**2.86**	0.35
7	Haptoglobin-related protein	P00739	HPR	2.56	2.14	2.09	4.7391	2.39E-05	4.6210	0.002931	**2.56**	0.39
8	GDH/6PGL endoplasmic bifunctional protein	O95479	H6PD	2.20	1.88	1.65	4.3812	0.000116	3.9340	0.007556	**1.97**	0.51
9	Leucine-rich alpha-2-glycoprotein	P02750	LRG1	1.91	1.64	1.46	4.7436	2.39E-05	4.6222	0.002931	**1.89**	0.53
10	Zinc finger protein 484	Q5JVG2	ZNF484	2.02	1.70	1.56	3.6403	0.000955	3.0199	0.030288	**1.89**	0.53
11	Translationally-controlled tumor protein	P13693	TPT1	1.83	1.62	1.43	3.9518	0.000399	3.3993	0.020228	**1.76**	0.57
12	Rho guanine nucleotide exchange factor 9	O43307	ARHGEF9	1.61	1.43	1.29	4.2335	0.000119	3.9239	0.007556	**1.73**	0.58
13	Putative inactive neutral ceramidase B	P0C7U1	ASAH2B	1.46	1.21	1.07	3.6373	0.000791	3.1017	0.026180	**1.61**	0.62
14	Serine/threonine-protein kinase	O95747	OXSR1	1.71	1.56	1.38	3.8332	0.000774	3.1111	0.026180	**1.61**	0.62
15	Alpha-1-antichymotrypsin	P01011	SERPINA3	1.36	1.14	1.02	4.6826	2.84E-05	4.5464	0.002931	**1.56**	0.64
16	Complement component C9	P02748	C9	1.24	1.05	0.94	4.693	2.80E-05	4.5535	0.002931	**1.50**	0.67
17	Immunoglobulin kappa constant	P01834	IGKC	1.13	0.96	0.91	3.6639	0.000681	3.1670	0.026180	1.48	0.68
18	Microtubule-associated protein 1A	P78559	MAP1A	1.22	1.14	1.01	5.0487	9.23E-06	5.0346	0.002931	1.46	0.69
19	DNA polymerase epsilon catalytic subunit A	Q07864	POLE	1.06	0.93	0.83	4.7148	2.56E-05	4.5916	0.002931	1.40	0.72
20	Girdin	Q3V6T2	CCDC88A	0.98	0.82	0.75	3.6491	0.00071	3.1485	0.026180	1.38	0.73
21	PTB domain-containing engulfment adapter protein 1	Q9UBP9	GULP1	1.07	0.89	0.80	3.6921	0.000767	3.1154	0.026180	1.38	0.73
22	Complement factor B	P00751	CFB	1.04	0.97	0.85	3.9297	0.000373	3.4282	0.020228	1.37	0.73
23	Coagulation factor IX	P00740	F9	0.78	0.65	0.68	3.6488	0.000708	3.1498	0.026180	1.29	0.78
24	Envoplakin	Q92817	EVPL	1.27	1.27	1.25	-3.7873	0.000534	3.2723	0.023912	0.72	1.39
