# Supplementary material for: Unveiling Distinct Proteomic Signatures in Complicated Crohn’s Disease That Could Predict the Disease Course
Source: Int J Mol Sci. 2023 Nov 30;24(23):16966. doi: 10.3390/ijms242316966 (PMC10707401; doi:10.3390/ijms242316966)
Supplement: Supplementary file 1 [file ijms-24-16966-s001.zip › Supplementary Table S3. Overview on the VIP scores PLS-DA, t-test and foldchanges of the 29 significantly different proteins among the groups.pdf]

Supplementary Table S3. Overview on the VIP scores PLS-DA, ANOVA and foldchanges of the 29 significantly different proteins among the groups

					VIPScore PLS-DA					Tukey's HSD					Fold change		
No.	Protein name	UniProtID	Gene	Comp. 1	Comp. 2	Comp. 3	f.value	p.value	FDR	Tukey's HSD	HC-B1	HC-B2B3	B1-B2B3	FC(B1vsHC)	FC(B1vsB2B3)	FC(B2B3vsB1)	FC(B2B3 vs HC)
1	Albumin	P02768	ALB	1.62	2.57	2.34	8.4211	0.000841	0.031999	B2B3-B1; HC-B2B3		x	x	0.7	**3.5**	0.3	0.90
2	Alpha-1-antichymotrypsin	P01011	SERPINA3	1.09	0.84	0.75	9.4252	0.000416	0.029702	B2B3-B1; HC-B2B3		x	x	1.3	0.8	1.3	1.04
3	Charged multivesicular body protein 3	Q9Y3E7	CHMP3	2.12	2.21	1.98	8.4396	0.00083	0.031999	B2B3-B1; HC-B2B3		x	x	0.6	**2.6**	0.4	0.88
4	Complement component C9	P02748	C9	1.19	0.9	0.8	8.1334	0.001034	0.034202	HC-B2B3		x		1.4	0.9	1.1	1.04
5	Complement factor I	P05156	CFI	0.51	0.4	0.35	7.5318	0.001602	0.042041	HC-B2B3		x		1.1	0.8	1.2	1.02
6	DNA polymerase epsilon catalytic subunit A	Q07864	POLE	0.9	0.67	0.61	9.1396	0.000507	0.029702	B2B3-B1; HC-B2B3		x	x	1.3	0.8	1.2	1.04
7	Dynein axonemal heavy chain 7	Q8WXX0	DNAH7	1.31	1.97	1.9	7.8533	0.001266	0.035687	HC-B1; HC-B2B3	x	x		0.7	2.7	0.4	0.90
8	Epididymal secretory glutathione peroxidase	O75715	GPX5	2.47	3.02	2.76	10.998	0.000144	0.026227	HC-B1; HC-B2B3	x	x		0.6	5.1	0.2	0.75
9	GDH/6PGL endoplasmic bifunctional protein	O95479	H6PD	1.88	1.44	1.28	11.397	0.000111	0.026227	B2B3-B1; HC-B2B3		x	x	1.6	0.7	1.4	1.11
10	Haptoglobin	P00738	HP	4.7	3.7	3.35	8.1782	0.001001	0.034202	HC-B1; HC-B2B3	x	x		7.9	1	1	1.16
11	Haptoglobin-related protein	P00739	HPR	2.3	1.86	2	8.0596	0.00109	0.034569	B2B3-B1; HC-B2B3		x	x	2.2	0.8	1.3	1.10
12	High mobility group nucleosome-binding domain-containing protein 5	P82970	HMGN5	0.53	0.8	0.72	8.9428	0.000581	0.029702	HC-B1; HC-B2B3	x	x		0.9	0.7	1.5	1.03
13	Immunoglobulin kappa constant	P01834	IGKC	0.69	0.6	0.58	8.841	0.000624	0.029702	HC-B1; HC-B2B3	x	x		1.3	0.7	1.3	1.04
14	Leucine-rich alpha-2-glycoprotein	P02750	LRG1	1.36	1.06	0.94	14.017	2.17E-05	0.016524	B2B3-B1; HC-B2B3		x	x	1.5	0.6	**1.6**	1.07
15	Lumican	P51884	LUM	1.42	1.59	1.42	8	0.001138	0.034648	HC-B1; HC-B2B3	x	x		0.7	1.8	0.5	0.93
16	Lysine-specific demethylase 3A	Q9Y4C1	KDM3A	1.57	1.71	1.54	7.588	0.001537	0.041781	B2B3-B1; HC-B2B3		x	x	0.7	**2.2**	0.4	0.92
17	Microtubule-associated protein 1A	P78559	MAP1A	1.43	1.12	1	8.2365	0.00096	0.034202	B2B3-B1; HC-B2B3		x	x	1.4	1	1	1.05
18	Phosphatidylinositol 4_5-bisphosphate 3-kinase catalytic subunit gamma isoform	P48736	PIK3CG	1.9	2.19	2.07	8.5577	0.000763	0.031999	B2B3-B1; HC-B2B3		x	x	0.6	**3.6**	0.3	0.87
19	Phosphatidylinositol 5-phosphate 4-kinase type-2 alpha	P48426	PIP4K2A	0.49	0.62	0.58	7.945	0.001185	0.034672	HC-B2B3		x		1.1	0.8	1.3	1.03
20	Plasma serine protease inhibitor	P05154	SERPINA5	1.79	1.72	1.56	9.1968	0.000487	0.029702	B2B3-B1; HC-B2B3		x	x	0.7	**2.1**	0.5	0.90
21	Plexin-A2	O75051	PLXNA2	1.97	2.28	2.1	10.219	0.000242	0.029028	B2B3-B1; HC-B2B3		x	x	0.7	**3**	0.3	0.86
22	PTB domain-containing engulfment adapter protein 1	Q9UBP9	GULP1	0.75	0.65	0.6	9.3935	0.000425	0.029702	B2B3-B1; HC-B2B3		x	x	1.2	0.8	1.3	1.04
23	Putative inactive neutral ceramidase B	P0C7U1	ASAH2B	1.09	0.93	0.85	9.8761	0.000305	0.029028	HC-B2B3		x		1.4	0.7	1.4	1.06
24	Serine/threonine-protein kinase OSR1	O95747	OXSR1	2.15	1.67	1.48	10.728	0.000172	0.026227	HC-B2B3		x		1.7	1.1	0.9	1.07
25	Serum amyloid A-1 protein	P0DJI8	SAA1	1.3	1.79	1.75	8.8854	0.000605	0.029702	B2B3-B1; HC-B2B3		x	x	2.6	0.6	**1.6**	1.11
26	Tetratricopeptide repeat protein 9A	Q92623	TTC9	1.9	2.57	2.45	8.4334	0.000834	0.031999	HC-B1; HC-B2B3	x	x		0.6	4.1	0.2	0.86
27	Transgelin-2	P37802	TAGLN2	1.73	1.37	1.23	8.9027	0.000598	0.029702	HC-B1; HC-B2B3	x	x		1.4	0.9	1.1	1.08
28	Translationally-controlled tumor protein	P13693	TPT1	1.64	1.24	1.12	11.822	8.45E-05	0.026227	HC-B1; HC-B2B3	x	x		1.5	0.7	1.4	1.08
29	WD repeat-containing protein 31	Q8NA23	WDR31	6.73	5.05	4.6	9.9393	0.000292	0.029028	B2B3-B1; HC-B2B3		x	x	4.7	0.4	**2.7**	**1.50**
