# Supplementary figures and images for: Unveiling Distinct Proteomic Signatures in Complicated Crohn’s Disease That Could Predict the Disease Course
Source: Int J Mol Sci. 2023 Nov 30;24(23):16966. doi: 10.3390/ijms242316966 (PMC10707401; doi:10.3390/ijms242316966)

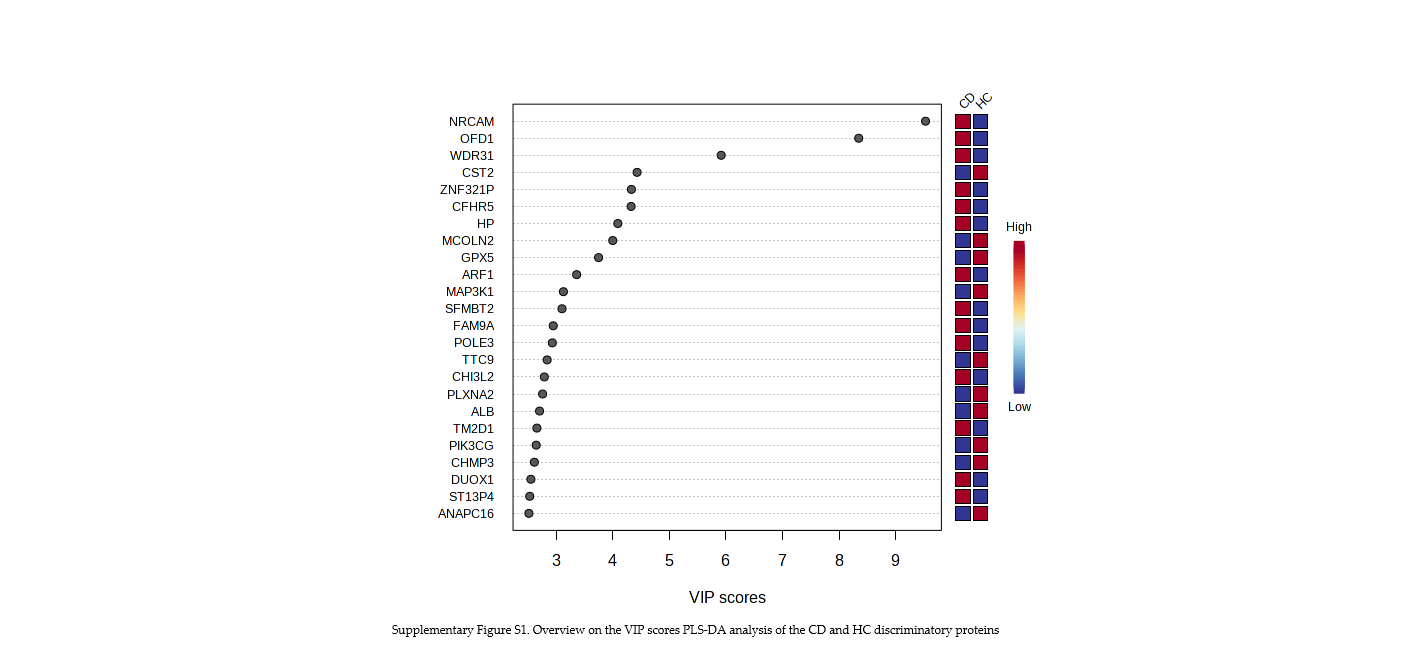

Supplement: Supplementary file 1 [file ijms-24-16966-s001.zip › Supplementary Figure S1. Overview on the VIP scores PLS-DA analysis of the CD and HC discriminatory proteins.png]
